# An Origin of Cooperative Oxygen Binding of Human Adult Hemoglobin: Different Roles of the α and β Subunits in the α_2_β_2_ Tetramer

**DOI:** 10.1371/journal.pone.0135080

**Published:** 2015-08-05

**Authors:** Shigenori Nagatomo, Yukifumi Nagai, Yayoi Aki, Hiroshi Sakurai, Kiyohiro Imai, Naoki Mizusawa, Takashi Ogura, Teizo Kitagawa, Masako Nagai

**Affiliations:** 1 Department of Chemistry, University of Tsukuba, Tsukuba, Ibaraki, Japan; 2 Research Center for Micro-Nano Technology, Hosei University, Koganei, Tokyo, Japan,3 School of Health Sciences, College of Medical, Pharmaceutical and Health Sciences, Kanazawa University, Kanazawa, Ishikawa, Japan,4 Department of Frontier Biosciences, Hosei University, Koganei, Tokyo, Japan,5 Picobiology Institute, Graduate School of Life Science, University of Hyogo, RSC-UH Leading Program Center, Sayo, Sayo-gun, Hyogo, Japan; Università degli Studi di Milano, ITALY

## Abstract

Human hemoglobin (Hb), which is an α_2_β_2_ tetramer and binds four O_2_ molecules, changes its O_2_-affinity from low to high as an increase of bound O_2_, that is characterized by ‘cooperativity’. This property is indispensable for its function of O_2_ transfer from a lung to tissues and is accounted for in terms of T/R quaternary structure change, assuming the presence of a strain on the Fe-histidine (His) bond in the T state caused by the formation of hydrogen bonds at the subunit interfaces. However, the difference between the α and β subunits has been neglected. To investigate the different roles of the Fe-His(F8) bonds in the α and β subunits, we investigated cavity mutant Hbs in which the Fe-His(F8) in either α or β subunits was replaced by Fe-imidazole and F8-glycine. Thus, in cavity mutant Hbs, the movement of Fe upon O_2_-binding is detached from the movement of the F-helix, which is supposed to play a role of communication. Recombinant Hb (rHb)(αH87G), in which only the Fe-His in the α subunits is replaced by Fe-imidazole, showed a biphasic O_2_-binding with no cooperativity, indicating the coexistence of two independent hemes with different O_2_-affinities. In contrast, rHb(βH92G), in which only the Fe-His in the β subunits is replaced by Fe-imidazole, gave a simple high-affinity O_2_-binding curve with no cooperativity. Resonance Raman, ^1^H NMR, and near-UV circular dichroism measurements revealed that the quaternary structure change did not occur upon O_2_-binding to rHb(αH87G), but it did partially occur with O_2_-binding to rHb(βH92G). The quaternary structure of rHb(αH87G) appears to be frozen in T while its tertiary structure is changeable. Thus, the absence of the Fe-His bond in the α subunit inhibits the T to R quaternary structure change upon O_2_-binding, but its absence in the β subunit simply enhances the O_2_-affinity of α subunit.

## Introduction

We uptake O_2_ (oxygen) in a lung through breathing. Oxygen molecules incorporated are transported to tissues by hemoglobin (Hb) in blood and are mostly reduced to water by cytochrome oxidase at mitochondria in order to synthesize ATP, that is, to create energy [1,2]. This activity is vital for life maintenance. Human adult Hb (Hb A) is a tetramer protein composed of two α (141 residues) and two β (146 residues) subunits (α_2_β_2_), each of which contains a single heme [1–3]. The incorporated O_2_ molecule is bound to Fe(II) ion of heme. Accordingly, a single Hb molecule can bind four O_2_ molecules. The O_2_ binding/release by heme is based on chemical equilibrium. Generally in chemical equilibrium, the concentrations of O_2_-bound forms are proportional to partial pressure of O_2_ in the absence of interactions among O_2_-binding sites. The partial pressures of O_2_ in lung and tissues are *ca*.100 and 40 mmHg, respectively. For effective O_2_ transfer under such a small difference in partial pressures, sensitive O_2_ binding/release should occur upon a small change of O_2_ pressure. In other words, nonlinear dependence of the concentrations of O_2_-bound forms on partial pressure of O_2_ is desirable [3].

Native Hb A which can bind four O_2_ molecules satisfies this requirement by switching O_2_-affinity from low- to high-state during oxygenation [3–6] and exhibits a characteristic property called cooperativity [3]. The Adair model [7], a mathematical description of cooperative oxygen binding in terms of the successive four binding constants, gives the following picture: when the first O_2_ molecule binds to deoxygenated Hb A (deoxyHb A), the four binding sites are equivalent with low affinity, but when the second one binds to the single-O_2_ bound form, the affinity of the remaining three sites are higher than the affinity for the first one. More changes toward the same direction occur for the third and fourth steps of O_2_ binding. Here, the four O_2_ binding sites in the α_2_β_2_ tetramer, which are separated by 2–2.5 nm, are not likely to have some direct communication (heme-heme interaction) upon O_2_ binding. Therefore, heme-heme interactions, an indirect interaction among hemes, have been considered to be mediated by conformational changes of the protein moieties that are associated with oxygenation [3]. Recent studies indicate that there is no subunit communication as far as the protein conformation is locked, as demonstrated with Hb A in a crystal or encapsulated in silica gels [8–11]. Historically, this phenomenon has been interpreted in terms of MWC (Monod-Wyman-Changeux) model [12], which assumes a transition between two quaternary states, i.e., a switch from a low to a high affinity state during oxygenation [3–6]. In the real solution state hemoglobin behaves as if the heme-heme interactions really occurred because oxygenation is accompanied by transitions between the two quaternary states.

Hill first formalized this cooperativity in 1913 [13], which is known as Hill plot [1–3,13]. The cooperativity of Hb A appears only in α_2_β_2_ hetero-tetramer. When Hb A is separated into subunits, α and β form homo-dimer (α_2_) and homo-tetramer (β_4_), respectively, but they do not exhibit the property of cooperative O_2_ binding. An αβ dimer has high O_2_ affinity but exhibits no cooperativity in O_2_ binding [3].

This property of Hb A has been extensively investigated as a general model of allosteric proteins [12,14]. MWC model [12] is known as two state model, or concerted model, in which it assumes that hemoglobin molecule takes one state of two states corresponding to differences of oxygen affinity. Thus far the MWC model has described a lot of properties of hemoglobin cooperativity in terms of absence of direct subunit-subunit communications and control of oxygen affinity by quaternary transitions. This model ascribes the heterotropic effects caused by protons, CO_2_, 2,3-BPG (bisphosphoglycerate) etc. to shifts of the allosteric equilibrium between the two quaternary structures. However, Imai [3] showed that these heterotropic effects cannot be realized without assuming that the oxygen affinity of either quaternary structure is influenced by heterotropic effectors bound, and he introduced a third affinity state, S, to explain the heterotropic effects in the framework of the control of oxygen affinity by quaternary transitions. Recently, a tertiary two state model (TTS model) was proposed to solve this inconsistence to account for heterotropic effects with the essence of MWC model conserved. In this model two tertiary structures (t and r) were incorporated into each quaternary structure [8–11]. By assuming that oxygen affinity of Hb A is controlled more by tertiary structure rather than quaternary structure [8–11], the TTS model can interpret the results of O_2_ binding in solution, gel states and single crystal consistently. On the contrary, KNF (Koshland-Némethy-Filmer) model [14] is suitable to describe each step of oxygen binding but lacks a systematic view regarding structural changes.

Each subunit of the hetero-tetramer has one protoheme (protoporphyrin-IX) coordinatively bound to an imidazole side chain of a histidine residue of the F helix (His F8). X-ray crystallographic studies of Hb A have demonstrated the presence of two distinct quaternary structures which correspond to the low-affinity (tense or T) and high-affinity (relaxed or R) states. Typical structures of the T and R states are observed for the unliganded form (deoxyHb A) and O_2_- or CO-bound form (oxyHb A or COHb A), respectively [4–6].

It is considered that a quaternary structure change from the T to R structure upon ligand binding is triggered by a movement of the iron-proximal histidine (Fe-His) bond [4–6]. Many studies have tried to elucidate the relationship between the Fe-His bond and quaternary structure change [15–45], some of which have pointed out the appreciable difference in the Fe-His bonds of the α and β subunits [25–45]. For instance, a recent high resolution X-ray crystallographic analysis [46] showed a slightly longer Fe-His bond-length for the α subunits in crystal; the Fe-His bond-lengths in each of the α and β subunits are (220, 221) pm and (216, 219) pm in deoxyHb A (T structure), respectively. The Fe-His stretching mode (ν_Fe-His_) is usually observed at 215 cm^-1^ for deoxyHb A under physiological conditions [47–51]. Although this wavenumber of ν_Fe-His_ is regarded as a marker of the T state [47–51], the observed Fe-His Raman band contains contributions from both the α and β subunits. Studies of valency-hybrid Hbs, including Hb M Boston (α^Μ^Fe^3+^-βFe^2+^), Hb M Milwaukee (αFe^2+^-β^Μ^Fe^3+^), α(Fe^3+^-CN)β(Fe^2+^-deoxy) and α(Fe^2+^-deoxy)β(Fe^3+^-CN), demonstrated a clear difference in the ν_Fe-His_ frequencies between the α and β subunits. It is now established that the ν_Fe-His_ modes of deoxyHb in the T state are located at 203 − 207 and 217 − 220 cm^-1^ for the α and β subunits, respectively [50], being consistent with the longer Fe-His bond-length in the α rather than β subunits in crystal [46]. Recently, Jones *et al*. has reported that the evolution of ν_Fe-His_ from R to T after CO photodissociation is faster for β(Fe-His) than for α(Fe-His) [45]. Thus it is suggested strongly that the properties of the Fe-His bond are different between the α and β subunits.

To investigate the different roles of the Fe-His bond between the α and β subunits in the regulation of oxygen affinity, many studies have been carried out using metal- or valency-hybrid-Hbs, in which the Fe-His bond in either the α or β subunit is not ordinary [25–50]. Thus far, we have elucidated some of the relations between the changes in the α_1_-β_2_ subunit contacts upon ligand binding and the magnitude of the strain in the Fe-His bond of the respective α or β subunits by using ultraviolet resonance Raman (UVRR) spectra for the former and visible RR spectra for the latter [35,37,41,52]. In the absence of the Fe-His bond in the α subunit, ligand binding to β heme causes incomplete quaternary structure change [35,37,41,52], and thus the O_2_ affinity of the β subunits remains very low, as seen from the *p*_50_ values of 65, 197, and 27.5 mmHg for α(Fe-NO)β(Fe-deoxy) [34], α(Ni)β(Fe-deoxy) [53], and Hb M Boston at pH 7 [52], respectively. It is noted for these hybrid Hbs that there is no covalent bond between the proximal His and the heme in the α subunits. Regrettably, however, all these studies had a common weak point, that is, both the metal- and valency-hybrid Hbs are unable to adopt the fully ligand-bound form, because Fe^3+^-heme or metal-substituted heme cannot bind ligands such as O_2_ or CO. Therefore, in regard to the number of ligands bound to a single Hb molecule, the hybrid Hbs have proven inadequate for investigating the ligand-bound form of Hb A.

**Fig 1:**
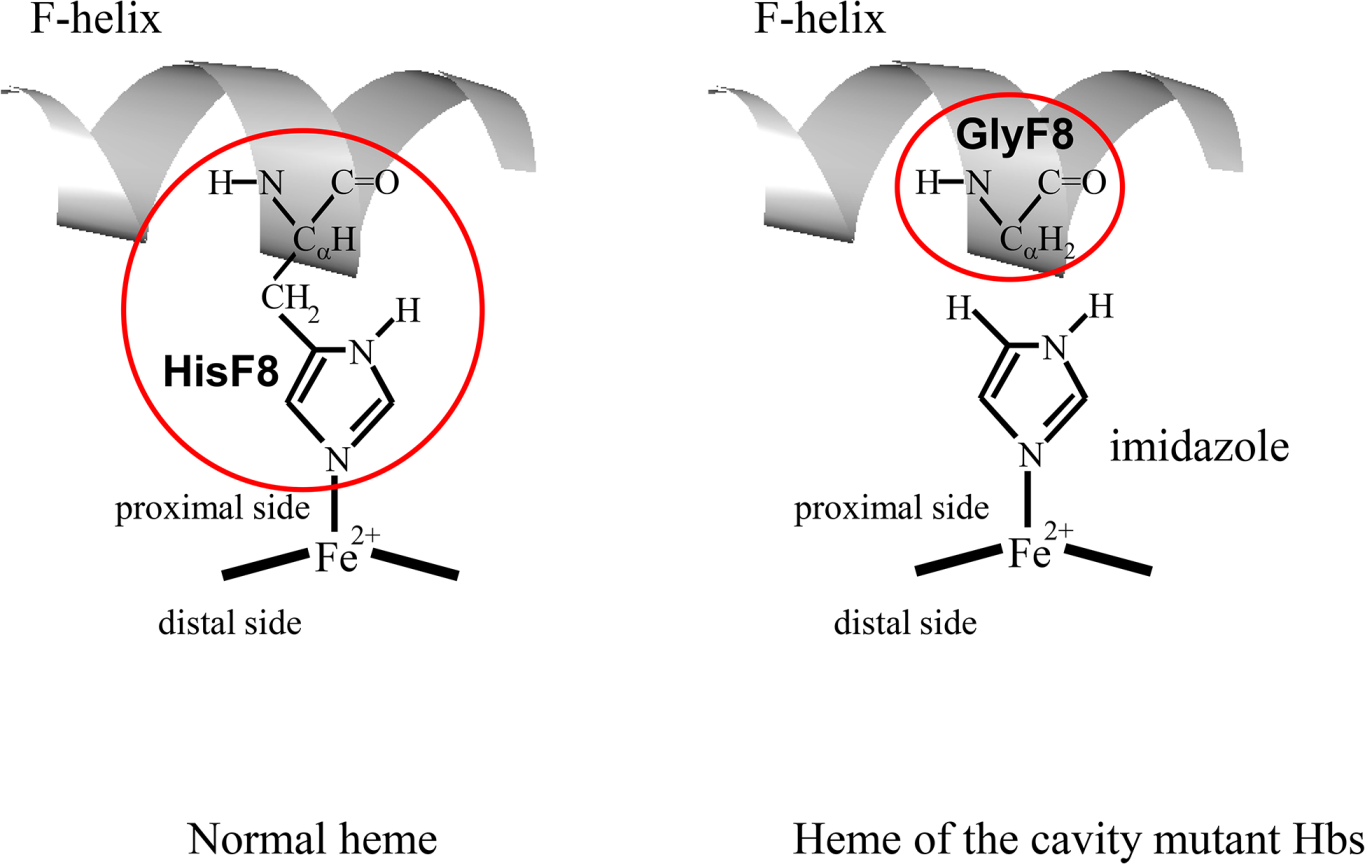
Schematic presentation of the normal heme (left) and the cavity mutant heme (F8His→Gly) (right). The crystal structure of the cavity mutant Mb, rMb(H93G), determined in the presence of imidazole, revealed that an imidazole molecule is bonded to the heme iron on the proximal side, as shown here (Barrick, D. *et al*., *Biochemistry* **1994**, *33*, 6546−6554). Atomic coordinates of 2DN2 (ref. 46) were used about description of ribbon model of F-helix.

Therefore, we planned to prepare cavity mutant Hbs, rHb(αH87G) and rHb(βH92G), which can bind four O_2_ molecules per one Hb molecule. The cavity mutant Hbs were first constructed by Ho’s group, and its ligand binding properties were examined using *n*-butylisocyanide and ^1^H NMR spectra [54]. As illustrated in Fig 1, the cavity mutant Hbs have similar properties to hybrid Hbs regarding the absence of the Fe-His bond in either the α or β subunit, for which a movement of the Fe-Im bond concomitant with ligand binding is not directly communicated to the F-helix [54]. In addition, the cavity mutant Hbs can take the fully ligand-bound form, because O_2_ or CO can bind to the imidazole-bound heme, and therefore, the effects of the interactions between bound ligand and the protein in the distal pocket are maintained.

In the present study attention is focused on the differences between the α and β subunits in terms of the roles of the proximal His for the regulation of O_2_ binding properties. The present results indicate that the detachment of a heme from F-helix in the α subunits inhibits the quaternary structure change from T to R upon ligand binding, being compatible with the results from hybrid Hbs, and that a similar detachment in the β subunits enhances the O_2_ affinity of the α subunit, which was not previously reported. It became clear that the high affinity of the α subunit was caused by the quaternary structure changes from T to R at the α_1_-β_2_ contacts, while the quaternary structures in the *C*-terminal regions are retained in T.

## Materials and Methods

### Preparation and Purification of Hemoglobins

Hb A was purified from human hemolysate by preparative isoelectric focusing [55]. Human hemolysate was prepared from concentrated red cell gifted from Japanese Red Cross Kanto-Koshinetsu Block Blood Center. Cavity mutant hemoglobins were prepared by site-directed mutagenesis in *E*. *coli*. The Hb A expression plasmid pHE7 [56], containing human α- and β-globin genes and the *E*. *coli* methionine aminopeptidase gene, was kindly provided by Professor Chien Ho of Carnegie Mellon University. The plasmids for rHb(αH87G) and rHb(βH92G) were produced using an amplification procedure for closed circular DNA *in vitro* [57] and transformed into *E*. *coli* JM109. The *E*. *coli* cells harboring the plasmid were grown at 30°C in TB medium [56]. The expression of recombinant hemoglobin (rHb) was induced by adding isopropyl β-thiogalactopyranoside along with 10 mM imidazole. The culture was then supplemented with hemin (30 μg/ml) and glucose (15 g/liter), and the growth was continued for 5 h at 32°C. The cells were harvested by centrifugation and stored under freezing conditions at -80°C until needed for purification. Recombinant Hbs were purified according to the methods described before [58], except for the fact that all procedures were carried out along with 10 mM imidazole. The rHb(βH92G) was separated into two main components on 2^nd^ Q Sepharose column chromatography. The elution profile of the Sephadex G-75 column showed that the former components were dimeric and the latter tetrameric. We used the tetrameric component for rHb(βH92G).

### Oxygen Equilibrium Experiments

Oxygen equilibrium curves were determined by using an automatic oxygenation apparatus [3]. The spectrophotometer used was a double-beam spectrophotometer equipped with an integration sphere (model U-4000, Hitachi, Tokyo). The degree of oxygenation or deoxygenation of hemoglobin was monitored with absorbance at 560 nm. The temperature within the oxygenation cell was maintained at 25.0 ± 0.1°C. The hemoglobin concentration was 60 μM on a heme basis. The buffer used was 0.05 M bis-Tris buffer (pH 7.4) and 0.05 M Tris buffer (pH 7.9) both containing 0.1 M Cl^−^ and 5 mM imidazole. To minimize the autooxidation of hemoglobin during measurements, an enzymatic metHb-reducing system [59], together with catalase and superoxide dismutase [60,61], were added to each sample. The amount of autooxidized Hb (metHb) after O_2_ equilibrium measurement ranged from 0.9 to 5.5% of the total Hb. Oxygen affinity (*P*_50_) and cooperativity (Hill coefficient, *n*) were calculated from the best-fit stepwise Adair constants [7] that were evaluated from the equilibrium curve by a nonlinear least-squares method [3,62] or from linear regression analysis using Hill equation.

### ^1^H NMR measurements

The ^1^H NMR spectra were measured with a Bruker AVANCE 600 FT NMR spectrometer operating at the ^1^H frequency of 600 MHz at the OPEN FACILITY, the Research Facility Center for Science and Technology, the University of Tsukuba. The hemoglobin concentrations of Hb A, rHb(αH87G) and rHb(βH92G) were 1000, 800, and 500 μM on a heme basis in 0.05 M phosphate buffer (pH 7.0). In addition, rHb(αH87G) and rHb(βH92G) contained 10 mM imidazole. The deoxy and CO forms were prepared by adding sodium dithionite (1 mg/mL) to the oxy form after replacement of the inside air of the sample tube with N_2_ and CO, respectively. The spectra were obtained by a water suppression method by presaturation with approximately 4k–8k scans, a spectral width of 36 kHz (60 ppm), data points of 32k, a 90° pulse of a 12.0 μs, and recycle times of 0.5 s for the deoxy form and 1–3 s for the CO forms. Chemical shifts are given in ppm downfield from the sodium 2,2-dimethyl-2-silapentane-5-sulfonate, with the residual H^2^HO as an internal reference.

### UVRR Measurements

The excitation light at 229 nm (0.5 mW), which was obtained from an intra-cavity frequency-doubled Ar^+^ ion laser (Coherent, Innova 300C FRED), was introduced onto a sample from the lower front side of the spinning cell, which was a quartz NMR tube (Wilmad-LabGlass, 535-PP-9SUP, diameter = 5 mm). A 135° back-scattering geometry was adopted. The cell was spun using a hollow axis motor (Oriental Motor, BLU220A-5FR) at 160 rpm. The scattered light was collected and focused by two achromatic doublet lenses onto the entrance slit of a prism prefilter polychromator (Bunkoh-Keiki) in order to reject Rayleigh scattering. The prefilter was coupled to a 1-m single spectrograph (HORIBA JobinYvon, 1000M), equipped with a 200-nm-blazed holographic grating with 3600 grooves/mm. The dispersed light was detected with a UV-coated, liquid-nitrogen-cooled CCD detector (Roper Scientific, Spec10:400B/LN). The hemoglobin concentration was 200 μM (in heme) in a 0.05 M phosphate buffer (pH 7.0) containing 0.2 M SO_4_^2-^ as the internal intensity standard. In addition, rHb(αH87G) and rHb(βH92G) contained 10 mM imidazole. The deoxy and CO forms were prepared by adding sodium dithionite (1 mg/mL) to the oxy form after the replacement of the inside air of the sample tube with N_2_ and CO, respectively. The presented spectra are an average of 13 scans, each of which is the sum of 180 exposures, each exposure accumulating data for 10 sec. Raman shifts were calibrated with cyclohexane as a frequency standard and the frequency accuracy was ±1 cm^-1^ for well-defined Raman bands. The difference spectra were obtained so that the Raman band of SO_4_^2-^ (980 cm^-1^) was abolished. The integrity of the sample after exposure to the UV laser light was carefully confirmed by the visible absorption spectra measured before and after the UVRR measurements. When spectral changes were recognized, the Raman spectra were discarded. Visible absorption spectra were recorded with a Hitachi U-3310 spectrophotometer.

### Visible RR Measurements

Visible RR spectra were excited at 441.6 nm with a He/Cd laser (Kinmon Electric, model CD4805R), dispersed with a 1 m single polychromator (Ritsu Oyo Kogaku, model MC-100DG), and detected with a UV-coated, liquid-nitrogen-cooled CCD detector (Roper Scientific, LN/CCD-1100-PB/VISAR/1). The sample conditions of Hb are the same as those for UVRR. All measurements were carried out at room temperature with a spinning cell (1800 rpm). The laser power at the scattering point was 4.0 mW.

### CD Measurements

The measurements were carried out with a Jasco J-820 spectropolarimeter at 25°C using a (+)-10-camphor-sulfonic acid calibration. Absorption spectra were measured with a double-beam spectrophotometer (Hitachi, model U-3010). The Hb solution, the concentration of which was 45 μM (in heme) in 0.05 M phosphate buffer (pH 7) containing 5 mM imidazole, was measured at 25°C with a cell having a 2 mm light path-length. The scan speed was 20 nm/min, and 40 scans were averaged. To minimize the autoxidation of hemoglobin during measurement, an enzymatic metHb reducing system [59] together with catalase and superoxide dismutase were added to the sample solutions [60,61]. The CD spectra of the metHb reducing system with catalase and superoxide dismutase were measured separately under the same conditions and subtracted from the Hb spectra.

## Results

### Oxygen Binding Properties of Cavity Mutant Hemoglobins

**Fig 2:**
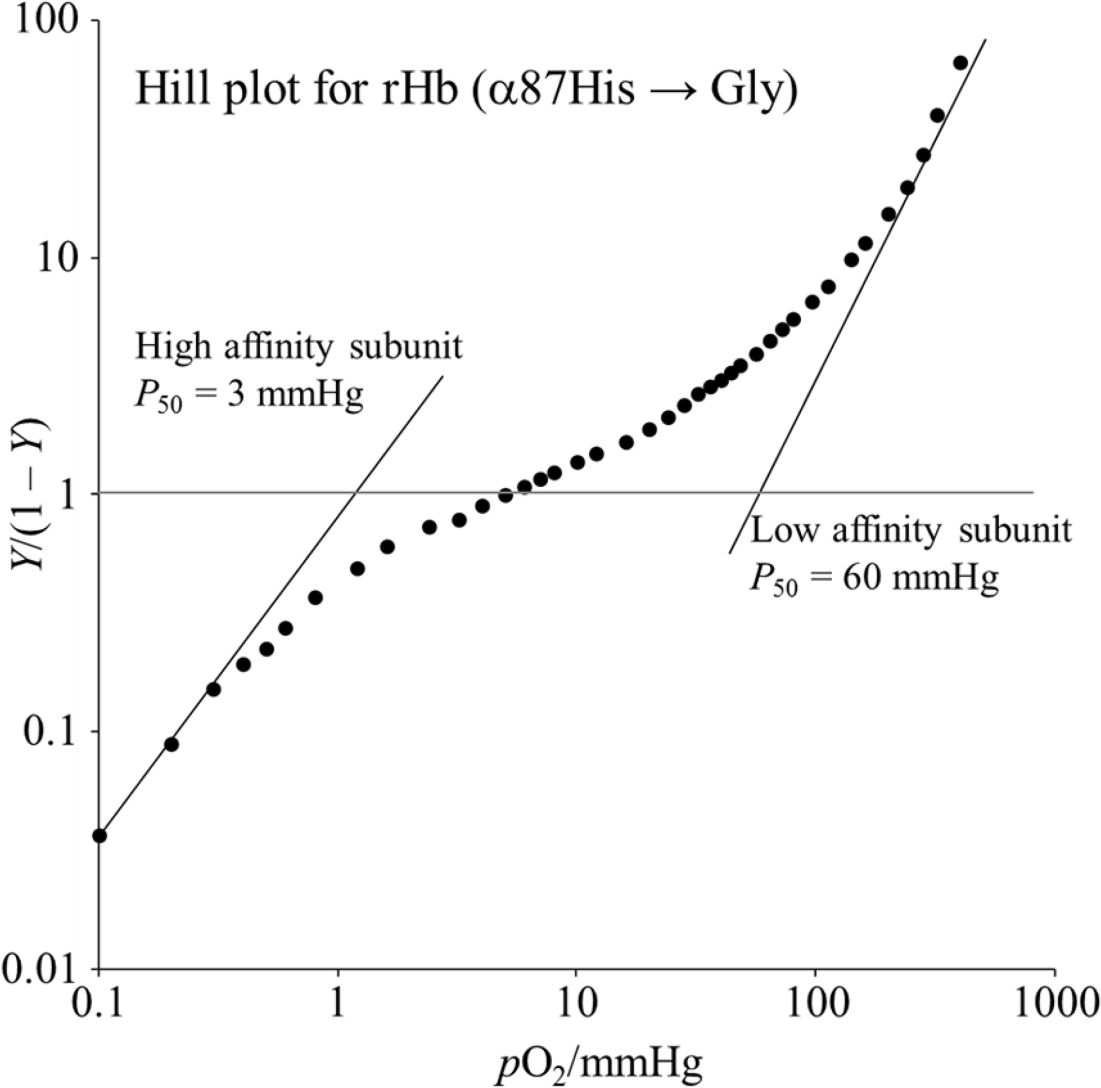
The Hill plot of oxygen binding by rHb(αH87G). The symbols are the observed points. *Y* is the fractional oxygen saturation and *p*O_2_ is the partial pressure of oxygen in millimeters of Hg. Asymptotic lines in the high and low affinity subunits are drawn by eye, and correspond to the *K*_1_ and *K*_4_ values of the four stepwise Adair constants, respectively. The hemoglobin concentration was 60 μM on a heme basis in 0.05 M bis-Tris buffer (pH 7.4) containing 0.1 M Cl^-^, 5 mM imidazole and a metHb reducing system. The temperature was set at 25°C.

**Fig 3:**
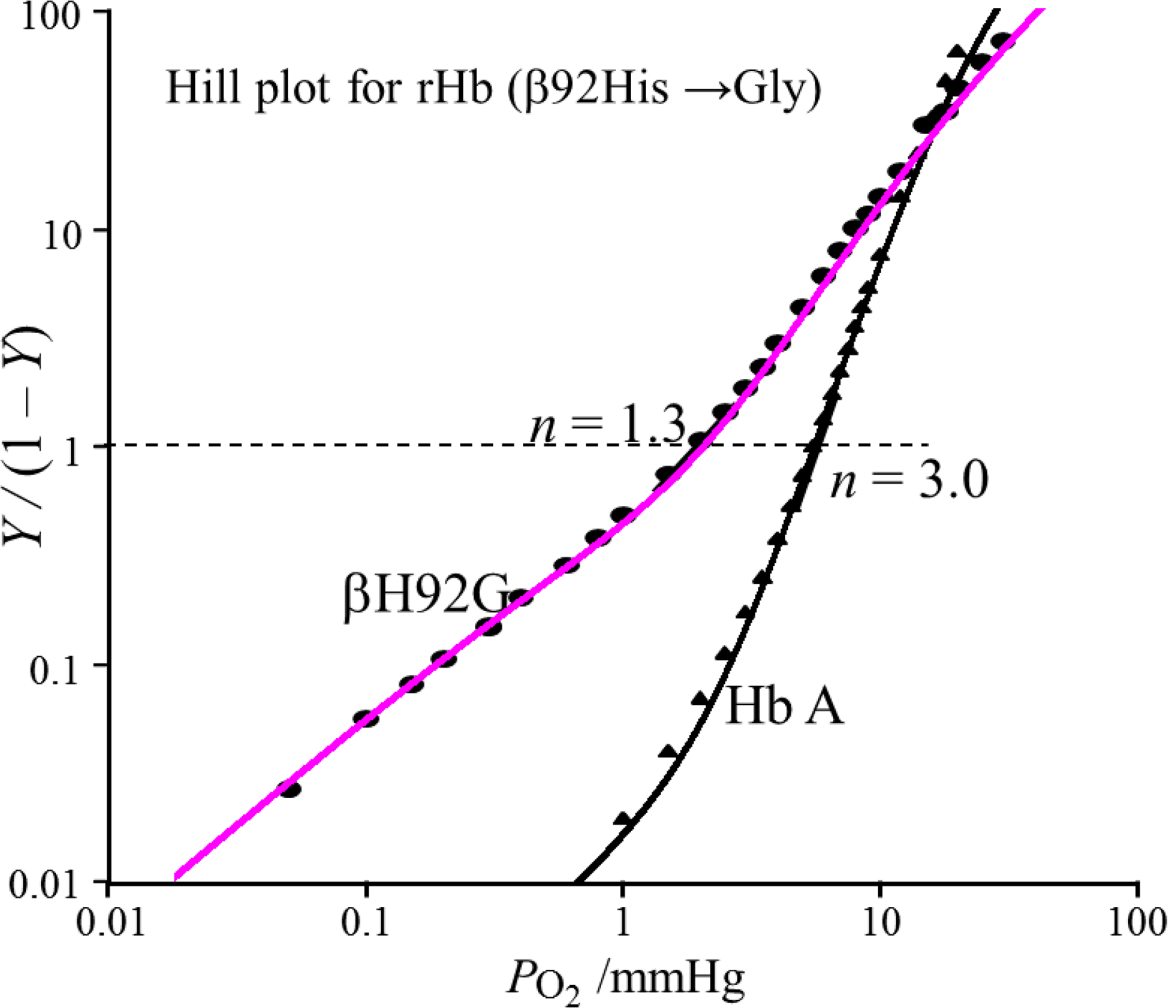
The Hill plots of oxygen binding by Hb A (▲) and rHb(αH92G) (●). *Y* and *p*O_2_ are as in Fig 2. The symbols are the observed points and lines were calculated from the best-fit values of the four stepwise Adair constants [3,7,62]. Adair constants, *K*_1_ and *K*_4_, used at fitting of rHb(βH92G) are 0.60 and 3.5 mmHg^-1^, those of Hb A are 0.014 and 8.0 mmHg^-1^, respectively. The hemoglobin concentration was 60 μM on a heme basis in 0.05 M bis-Tris buffer (pH 7.4) containing 0.1 M Cl^-^. In addition, rHb(βH92G) contained 5 mM imidazole and metHb reducing system. The temperature was 25°C.

**Fig 4:**
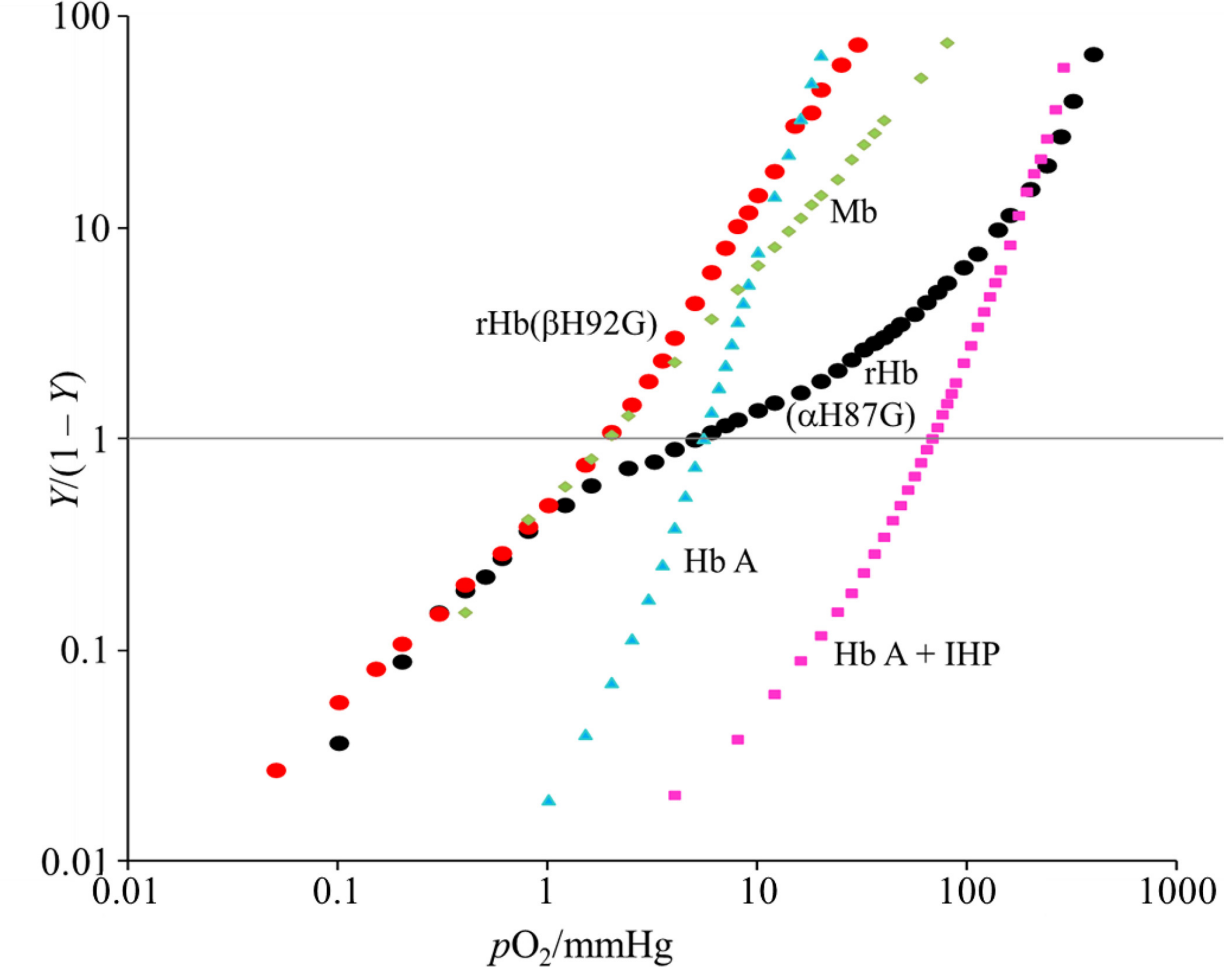
Hill’s plots of oxygen binding by Hb A, rHb(αH87G), rHb(βH92G) and sperm whale Mb. Hb A (-IHP) (blue closed triangle: ▲), Hb A (+IHP) (pink closed square: ■), rHb(αH87G) (black closed circle: ●), rHb(βH92G) (orange closed circle: ●) and sperm whale Mb (light green closed diamond: ◆). *Y* and *p*O_2_ are as in Fig 2. The symbols are the observed points. The hemoglobin concentration was 60 μM on a heme basis in 0.05 M bis-Tris buffer (pH 7.4) containing 0.1 M Cl^-^. In addition, rHb(αH87G) and rHb(βH92G) contained 5 mM imidazole and a metHb reducing system. The temperature was set at 25°C. IHP was added to a final concentration of 2 mM.

We expressed two mutant Hbs in *E*. *coli* and examined the effects of mutation on the O_2_ binding properties. As shown in Figs 2–4 and Table 1, both rHb(αH87G) and rHb(βH92G) exhibited O_2_ binding properties different from those of Hb A. Further, the O_2_ binding properties of these mutant Hbs were different from each other. We show UV-Vis absorption spectra of the mutant Hbs, rHb(αH87G) and rHb(βH92G), in S4 and S5 Figs, respectively.

**Table 1:** Oxygen binding properties of the cavity mutant Hbs, Hb A, and Mb.

	Conditions	*P*_50_	Hill’s *n*	*P*_50_^IHP^ */P*_50_^free^	δH^+^
rHb(αH87G)	pH 7.4	5.6 ± 0.1	0.45 ± 0.01	1.4 ± 0.1	-0.03
	pH 7.9	4.9 ± 0.1	0.35 ± 0.01		
	pH 7.4 + IHP	7.7 ± 0.2	0.31 ± 0.01		
rHb(βH92G)	pH 7.4	1.7 ± 0.1	1.2 ± 0.1	1.1 ± 0.1	-0.21
	pH 7.9	1.3 ± 0.1	1.2 ± 0.1		
	pH 7.4 + IHP	1.8 ± 0.1	0.78 ± 0.02		
Hb A	pH 7.4	5.5 ± 0.1	3.1 ± 0.1	12 ± 1	-0.45
	pH 7.9	4.3 ± 0.1	3.1 ± 0.1		
	pH 7.4 + IHP	66 ± 1	2.1 ± 0.1		
Mb	pH 7.4	2.0 ± 0.1	1.1 ± 0.1		

Experimental conditions: in 0.05 M bis-Tris (pH 7.4) or Tris (pH 7.9) containing 0.1 M Cl^-^; Hb conc., 60 μM on a heme basis; 25°C; with an enzymic metHb reducing system and 5 mM imidazole.

*n*: the Hill coefficient

*P*_50_: partial pressure of oxygen at half saturation (mmHg)

*P*_50_^IHP^ */ P*_*50*_^free^: ratio of *P*_50_ in the presence of IHP to *P*_50_ in its absence

δ*H* ^*+*^: the Bohr coefficience (= Δlog *P*_50_ / ΔpH)

Standard deviations of *P*_50_ and Hill coefficient, *n*, are shown by ± XX.

#### rHb(αH87G)

As shown in Fig 2, rHb(αH87G) gave a distinct biphasic O_2_ equilibrium curve and exhibited no Bohr effect (Table 1). This biphasic curve is reproduced by superimposing the curves of two independent subunits with widely different O_2_ affinities. This mechanism of biphasic shape in oxygen equilibrium curve of rHb(αH87G) is clearly distinguished from negative cooperativity which also gives an “apparent” biphasic curve. The calculated O_2_ affinities (*P*_50_) for the low- and high-affinity components of the biphasic curve were 60 mmHg and 3 mmHg, respectively. A similar biphasic curve is observed for silica gels encapsulated Hb A by Shibayama and Saigo [63].

The high affinity component is ascribable to the α subunits (detached heme) and the low affinity one to the β subunits (normal heme), because previous ^1^H NMR studies of rHb(αH87G) showed that the *n*-butylisocyanide binding to the detached α subunit was much faster than that to normal β subunits [54]. In addition to ^1^H NMR results [54], we have another support for high oxygen affinity of Fe-His cleaved α subunit. Oxygen affinity of the β subunit is low and their *P*_50_ values are *ca*. 60–200 mmHg, when the Fe-His (or Ni-His) is cleaved in the α subunit (for instance, Shibayama *et al*. [53] and Yonetani *et al*. [34]. Assuming that an effect of Fe-His cleavage is similar to that of a detachment between the F-helix and Fe-Im in the α subunit, low oxygen affinity of rHb(αH87G) can be attributed to oxygen affinity of the β subunit. Then, as a result, high oxygen affinity of rHb(αH87G) can be attributed to that of the α subunit. The oxygenation curve of rHb(αH87G) was successfully determined for the first time here. Further, it became clear that its oxygenation was not almost influenced by an allosteric effector, such as inositol hexaphosphate (IHP), in contrast to oxygenation of native Hb A. Oxygen equilibrium curves of rHb(αH87G) with and without IHP are shown in S6 Fig or S1 File. Although the effect of IHP on oxygen binding properties of rHb(αH87G) was very small, low oxygen affinity species of rHb(αH87G) was similar to Hb A with IHP.

#### rHb(βH92G)

The Hill plot of rHb(βH92G) was compared with that of Hb A in Fig 3, where the symbols denote the observed points and the smooth curves were calculated from the best-fit values of the four stepwise Adair constants [3,7,62]. Recombinant Hb(βH92G) exhibited little cooperativity (Hill’s *n* = 1.2 − 1.3), in contrast with *n* = 3.0 for Hb A, and its Bohr effect was half of that of Hb A (Table 1). The Hill plot of rHb(βH92G) was further compared with those of rHb(αH87G), Mb, and Hb A in the presence and absence of IHP in Fig 4. The O_2_ equilibrium curve of rHb(βH92G) demonstrated that its O_2_ affinity was high (*P*_50_ = 1.9 mmHg), similar to that of Mb [64]. It was not influenced by allosteric effectors such as IHP, as seen in rHb(αH87G), shown in S7 Fig or S1 File. Thus, although both rHb(βH92G) and swMb show similar oxygen affinities, rHb(βH92G) has quaternary structure but swMb has a tertiary structure only. Comparison of oxygen affinity of rHb(βH92G) with that of swMb seems important for understanding the increase of oxygen affinity in rHb(βH92G) in spite of its T quaternary structure in deoxy form. The equilibrium constant associated with the fourth oxygenation step, *K*_4_, of rHb(βH92G) exhibited a value similar to that of Hb A, but the equilibrium constant for the first oxygenation step, *K*_1_, was 30 times larger than that of Hb A (Fig 3). If we accept the idea that Fe-Im heme has a higher affinity than that of Fe-His heme in consonance with the previous ^1^H NMR results on ligand binding to rHb(αH87G) [54], the observed *K*_1_ and *K*_4_ indicates the affinity of the β(Fe-Im) and α(Fe-His) subunits, respectively. This means that the detachment of the F-helix from heme in the β subunits by a disconnection of the Fe-His bond causes an increase of O_2_ affinity in the α subunits whose hemes are attached to the F-helix, to the same degree as that of Mb.

### ^1^H NMR Spectra of Cavity Mutant Hemoglobins

**Fig 5:**
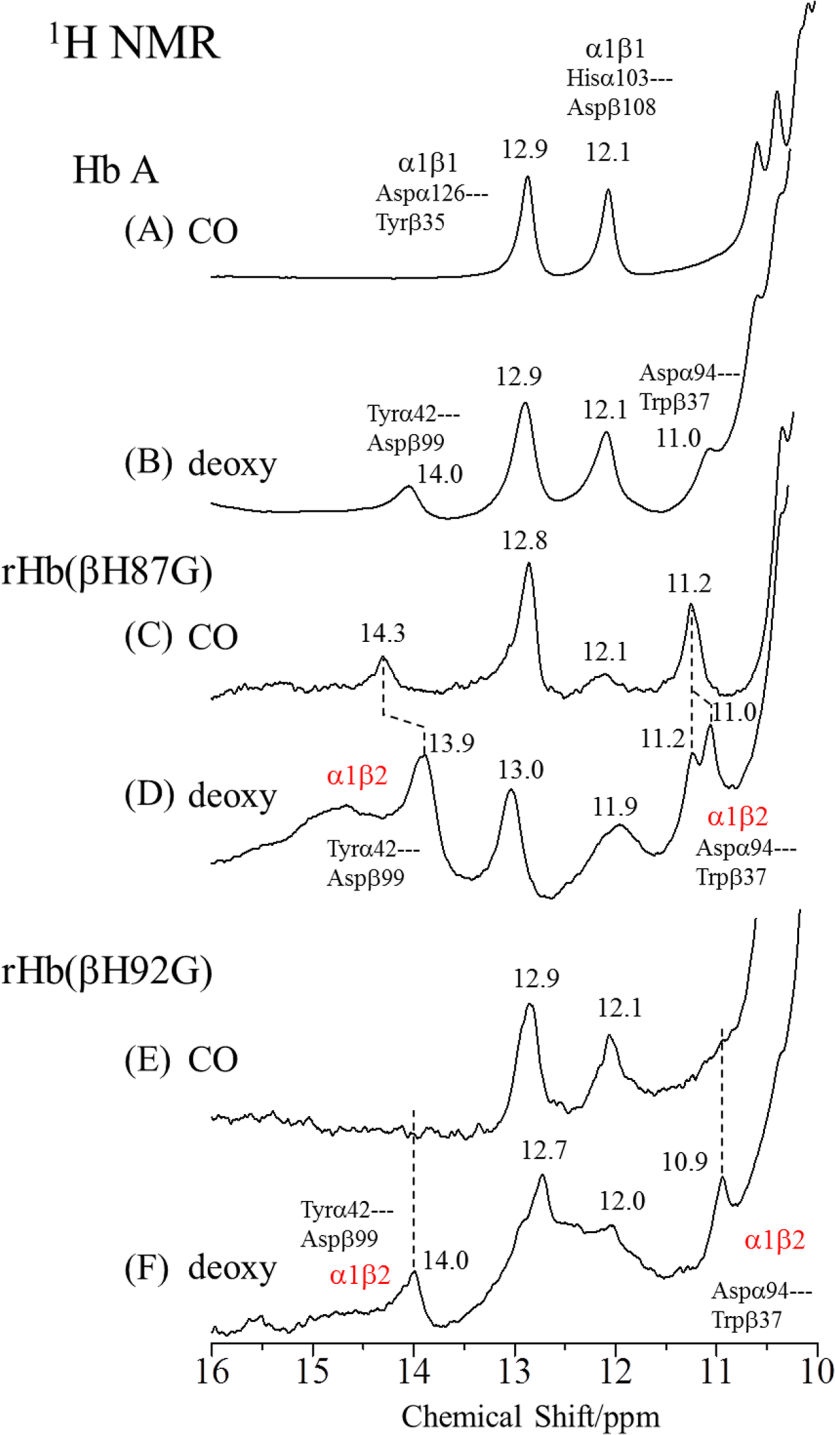
600 MHz ^1^H NMR spectra of Hb A, rHb(αH87G) and rHb(βH92G). Spectra are CO- and deoxyHb A (A, B), CO- and deoxy-rHb(αH87G) (C, D), and CO- and deoxy-rHb(βH92G) (E, F) between 10 and 16 ppm at pH 7.0 and 25°C. The hemoglobin concentrations of Hb A, rHb(αH87G) and rHb(βH92G) were 1 mM, 800 and 500 μM, respectively, on a heme basis in 0.05 M phosphate buffer (pH 7.0). In addition, rHb(αH87G) and rHb(βH92G) contained 10 mM imidazole.

Fig 5 compares the ^1^H NMR spectra of the deoxy- and CO-forms of rHb(αH87G) and rHb(βH92G) with those of Hb A in the frequency region between 16 and 10 ppm. In deoxyHb A (Fig 5B), four proton signals were resolved between 16 and 10 ppm and assigned as indicated in the figures [65]. These four protons were ascribed to hydrogen bonded ones at the α_1_-β_2_ and α_1_-β_1_ subunit-interfaces. As the signal around 14.0 ppm derived from the hydrogen bond between Tyrα42 and Aspβ99 at the α_1_-β_2_ interface can be observed only in the deoxy-form, this signal has generally been used as a marker of the T quaternary structure. The signal observed around 11.0 ppm has also been observed only for the deoxy-form, and therefore, was thought to arise from the hydrogen bond between Aspα94 and Trpβ37 at the α_1_-β_2_ interface, although there is a report that does not regard the 11.0 ppm signal as a T-marker [54]. The fact that both the 14.0 and 11.0 ppm signals are observed for rHb(αH87G) (Fig 5D) and rHb(βH92G) (Fig 5F) in the deoxy-form, similar to deoxyHb A, suggests that both rHb(αH87G) and rHb(βH92G) take the T quaternary structure in the deoxy-form.

In deoxy-rHb(βH92G), signals of 14.0 and 10.9 ppm characteristic of the T structure disappeared completely in the CO-form (Fig 5E). This suggests that CO-rHb(βH92G) is in the R quaternary structure, as reported previously [54]. In rHb(αH87G), on the other hand, not only signals of 14.3 ppm, but also of 11.2 ppm remained in the CO-form (Fig 5C), although in a slightly altered manner from that of the deoxy state. This indicates that CO-rHb(αH87G) is nearly in the T quaternary structure, as reported previously [54], except that the signal intensity of 11.2 ppm is noticeably stronger than that of deoxyHb A (Fig 5B). Thus, the protein structures are appreciably different between rHb(αH87G) and rHb(βH92G) in the CO-form, suggesting that CO-rHb(αH87G) adopts a T-like structure, but CO-rHb(βH92G) adopts the typical R structure for the α_1_-β_2_ contact. This observation indicates that the Fe-His bond in the α subunits is essential to the quaternary structure transition from the T to R at the α_1_-β_2_ contact regions.

The signals derived from the α_1_-β_1_ interface of rHb(αH87G) and rHb(βH92G) are also different from those of Hb A. The signals around 12.1 and 12.9 ppm of Hb A were assigned to the hydrogen bonded protons between Hisα103 and Aspβ108 and between Aspα126 and Tyrβ35, respectively [65–67]. Substitution of a Gly residue for the proximal His may induce tertiary structure changes, since these signals are thought to reflect the tertiary structure influencing the subunit contacts at the α_1_-β_1_ interface.

**Fig 6:**
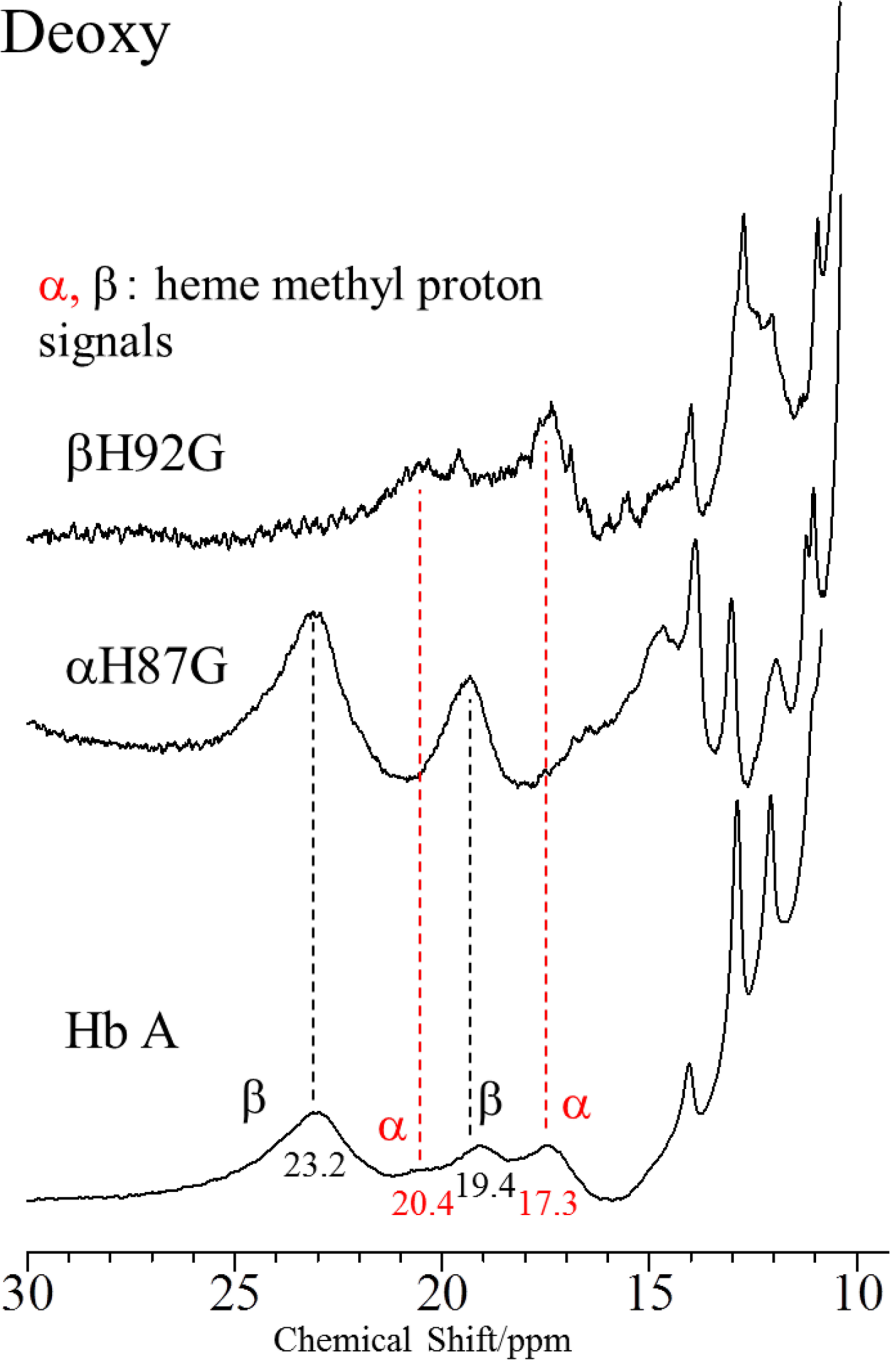
600 MHz ^1^H NMR spectra of Hb A, rHb(αH87G) and rHb(βH92G) in the deoxy-form. Spectra are between 10 and 30 ppm at pH 7.0 and 25°C. The hemoglobin concentrations of Hb A, rHb(αH87G) an rHb(βH92G) were 1 mM, 800 and 500 μM, respectively, on a heme basis in 0.05 M phosphate buffer (pH 7.0). In addition, rHb(αH87G) and rHb(βH92G) contained 10 mM imidazole.

The ^1^H NMR spectra in the heme methyl region (between 30 and 10 ppm) of Hb A, rHb(αH87G) and rHb(βH92G) in the deoxy-form are displayed in Fig 6, where the paramagnetically shifted heme methyl proton signals are observed at 23.2 and 19.4 ppm for the β heme and at 20.4 and 17.3 ppm for the α heme, respectively [26,27,65]. In the spectra of rHb(αH87G) only the methyl signals of heme derived from the normal β heme were observed at the same position as those of Hb A, and similarly in rHb(βH92G), only the methyl signals of heme from the normal α heme were observed. The fact that the chemical shifts of these heme methyl proton signals observed for rHb(αH87G) and rHb(βH92G) are very similar to those of deoxyHb A strongly suggests that the heme structures of the normal α subunits in rHb(βH92G) and normal β subunits in rHb(αH87G) have the Fe-His bond similar to native deoxyHb A. These paramagnetically shifted heme methyl proton signals disappeared upon CO binding to heme due to diamagnetism shown in S1 Fig or S1 File.

### UV Resonance Raman Spectra of Cavity Mutant Hemoglobins

**Fig 7:**
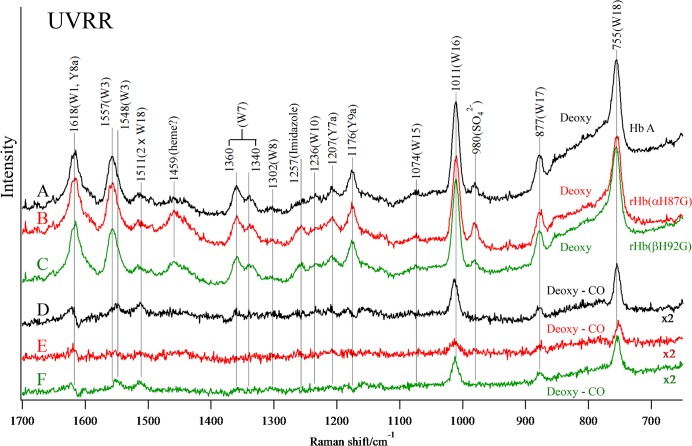
The 229-nm excited UVRR spectra of Hb A, rHb(αH87G) and rHb(βH92G). Spectra are deoxyHb A (A), deoxy rHb(αH87G) (B) and deoxy rHb(βH92G) (C), and the difference between Hb A (deoxy–CO) (D), rHb(αH87G) (deoxy–CO) (E) and rHb(βH92G) (deoxy–CO) (F). The hemoglobin concentration was 200 μM (in heme) in a 0.05 M phosphate buffer (pH 7.0) containing 0.2 M SO_4_^2-^ as the internal intensity standard. In addition, rHb(αH87G) and rHb(βH92G) contained 10 mM imidazole. The difference spectra were obtained so that the Raman band of SO_4_^2-^ (980 cm^-1^) could be abolished. The spectra shown are an average of 13 scans.

Fig 7 shows the 229-nm excited UVRR spectra of the deoxy-form and the deoxy-minus-CO difference spectra for Hb A (A, D), rHb(αH87G) (B, E) and rHb(βH92G) (C, F) in the frequency region from 1700 to 650 cm^-1^. The Raman bands of Tyr and Trp are marked by Y and W, respectively, followed by their mode number [68–71]. The band at 980 cm^-1^ arises from the SO_4_^2-^ ions added as an internal intensity standard. In the raw spectra, the intensities of the W3, W16, W17, and W18 bands of Trp are weaker in COHb A than in deoxyHb A, while the peak positions remain unaltered. Accordingly, positive peaks appear in the deoxy-minus-CO difference spectra. For the Tyr bands, however, the frequencies of the Y8a and Y9a bands of deoxyHb A are shifted lower in COHb A, and therefore, differential patterns appeared in the deoxy-minus-CO difference spectra (Fig 7D). These spectral differences have been ascribed to certain alterations in the hydrogen bonding and the surrounding hydrophobicity of the Trp and Tyr residues upon ligand binding [68–72]. Therefore, the deoxy-minus-CO difference spectrum of Hb A (Fig 7D) will serve hereafter as the standard for the T–R difference spectrum.

The deoxy-minus-CO difference spectrum of rHb(αH87G) (E) differs from that of Hb A as follows. The wavenumber shifts of the Y8a and Y9a bands are not observed at all and the peak intensities of Trp (especially W16 and W18) are smaller. This and the results from NMR (Fig 5) suggest that the quaternary structure change upon CO binding to rHb(αH87G) is much smaller than that of Hb A. Accordingly, the difference peak intensities of the Trp bands in Fig 7E might be attributed to tertiary structural changes. In contrast, the deoxy-minus-CO difference spectrum of rHb(βH92G) (F) was similar to that of Hb A (D). The intensity reduction of Trp bands upon CO binding of rHb(βH92G) was alike to Hb A and the wavenumber shifts of Tyr Y8a and Y9a bands, the extents of which were half of those in Hb A, are definitely present. This may suggest an incomplete quaternary structure change or tertiary structure changes for rHb(βH92G). To clarify whether these differences arose from the deoxy or CO-bound form, we examined the difference spectra between Hb A and rHb(βH92G) in both the deoxy- and CO-forms.

In the UVRR difference spectrum between rHb(βH92G) and Hb A in the deoxy-form shown in S2A Fig, the intensities of W16 and W18 of rHb(βH92G) are weaker than those of Hb A in the deoxy state (S2A and S2E Fig), but are alike in the CO-bound state (S2C and S2F Fig). S2E and S2F Fig are the difference spectra calculated from (S2A Fig–imidazole) and from (S2C Fig–imidazole), respectively. Thus, it became clear that the weaker intensities of the Trp bands in Fig 7F may be ascribed to the deoxy state. In the ^1^H NMR spectrum of rHb(βH92G) in the deoxy-form, the proton signal derived from the hydrogen bond between Trpβ37 and Aspα94 is clearly observed at 10.9 ppm (Fig 5F), although the upfield shift by 0.1 ppm may reflect a weaker hydrogen bond in deoxy-rHb(βH92G) than in deoxyHb A (11.0 ppm). Therefore, the weaker intensities of the Trp bands (W16 and W18) are attributed to residues other than Trpβ37, that is, Trpα14 or Trpβ15. Thus, it is suggested that there are small contributions from the tertiary structure change to the UVRR and ^1^H NMR spectra.

In the UVRR difference spectra between Hb A and those of the cavity mutant Hbs shown in S2 Fig, we noticed the presence of two additional bands at 1459 (a) and 1220 cm^-1^ (b). These peaks appear in a similar manner in rHb(αH87G) and rHb(βH92G) (S2A−S2D Fig). However, these peaks disappeared in the deoxy-minus-CO difference spectra, as shown by Fig 7E and 7F. Therefore, these peaks are present in the same way in both the deoxy and CO forms. Assuming these bands are derived from a heme, the bands of 1220 and 1459 cm^-1^ could be assigned to CH_2_ twisting and vinyl scissoring vibrations of (δ (= C_b_H_2_)), respectively [73]. This suggests the side-chain structure of Fe-Im heme is slightly different from that of His-heme (normal).

#### Visible Resonance Raman Spectra of Cavity Mutant Hemoglobins

**Fig 8:**
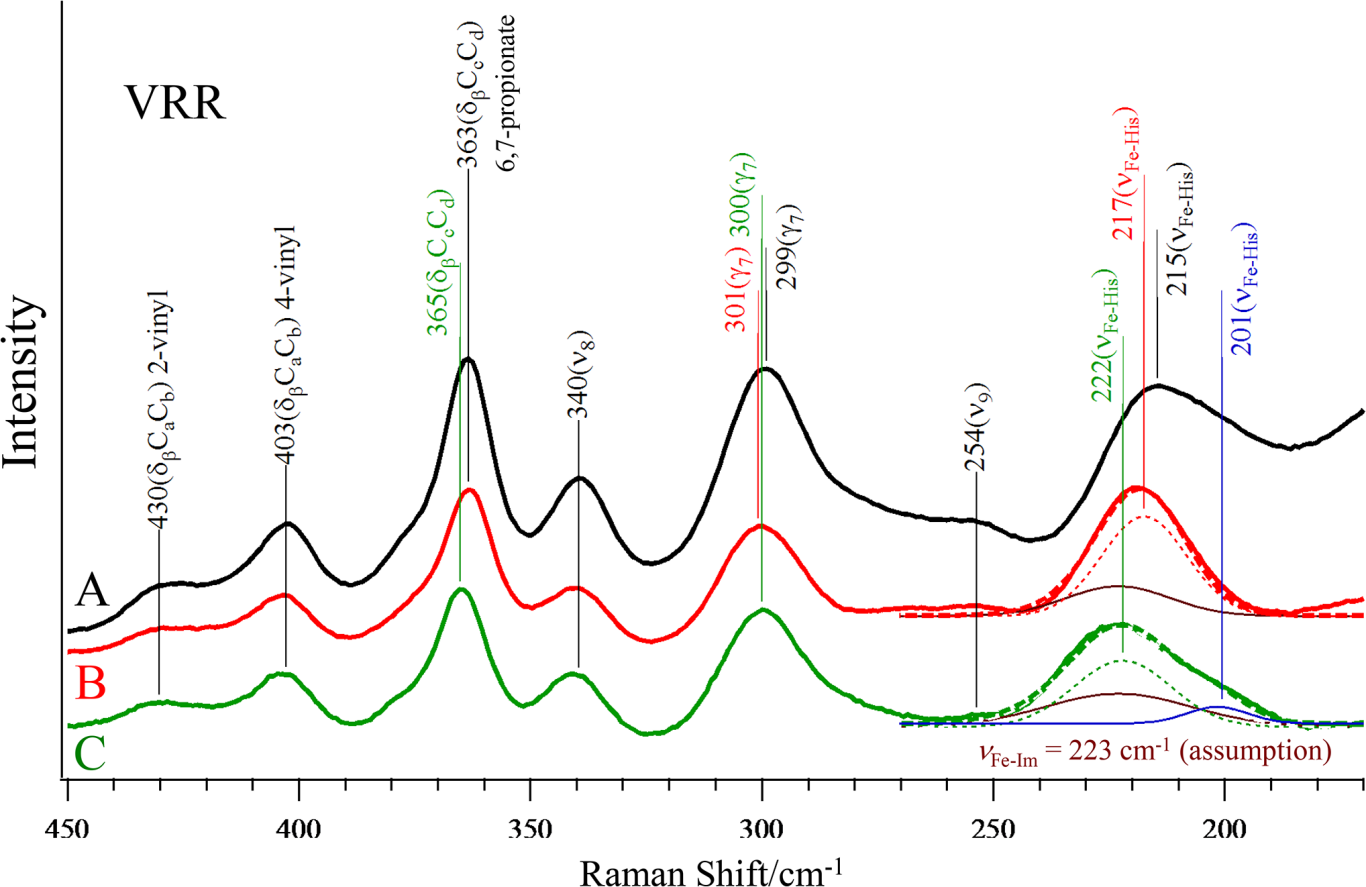
The 441.6-nm excited visible RR spectra of deoxyHb A (A), deoxy-rHb(αH87G) (B) and deoxy-rHb(βH92G) (C). The hemoglobin concentration was 200 μM (in heme) in a 0.05 M phosphate buffer, pH 7.0. In addition, rHb(αH87G) and rHb(βH92G) contained 10 mM imidazole. For rHb(αH87G), deconvoluted components, a ν_Fe-His_ and a ν_Fe-Im_ are indicated by a thin dotted red line and a brown solid line, respectively. For rHb(βH92G), deconvoluted components, a ν_Fe-His_ (high), a ν_Fe-His_ (low) and a ν_Fe-Im_ are indicated by a thin dotted green, a solid blue line and a solid brown line, respectively.

Fig 8 shows the 441.6 nm-excited resonance Raman spectra of Hb A (A), rHb(αH87G) (B) and rHb(βH92G) (C) in the deoxy form. Except for the bands around 220 cm^-1^ assignable to the Fe-His (ν_Fe-His_), or Fe-Im (ν_Fe-Im_) stretching and around 363 cm^-1^, the observed peak frequencies are almost the same among the three Hbs. The latter band is assigned to δ(C_β_C_c_C_d_)_6,7_, an in-plain bending mode of a propionate group [73]. This band appears at 363, 363, and 365 cm^-1^, for Hb A, rHb(αH87G) and rHb(βH92G), respectively. The δ(C_β_C_c_C_d_)_6,7,_ mode shifts to a higher wavenumber in rHb(βH92G) than Hb A and rHb(αH87G), suggesting a subtle disorder of the tertiary structure around the propionate side-chain of the Fe-Im heme of the β subunits in rHb(βH92G).

The bands assignable to the ν_Fe-His_ (215 cm^-1^ in deoxyHb A) were apparently observed at 218 and 222 cm^-1^ in deoxy-rHb(αH87G) and deoxy-rHb(βH92G), respectively (solid lines in Fig 8B and 8C). The apparent ν_Fe-His_ band of native deoxyHb A is expected to appear around 214–218 and 220–224 cm^-1^ for the typical T and R states, respectively [47–51], and therefore has been used often for a determination of whether a sample Hb in question is in the T state or R state. According to this criteria, rHb(αH87G) and rHb(βH92G) in the deoxy-form are in the T and R states, respectively. In practice, however, the ν_Fe-His_ frequency and intensity of deoxyHb A (Fig 8A) are different between the α and β subunits, so their intensity-weighted average is experimentally obtained [18,28,50].

The hemes in the α subunits of rHb(αH87G) and the β subunits of rHb(βH92G) have Fe-Im bonds, but no Fe-His bonds. It is known that isolated 2-methyl-imidazole-bound deoxy-Fe(II) protoporphyrin in solution gives a ν_Fe-Im_ band around 220 cm^-1^ [74]. Therefore, the ν_Fe-Im_ band should be overlapped with the ν_Fe-His_ band in the observed spectra of rHb(αH87G) and rHb(βH92G). Accordingly, the apparent ν_Fe-His_ band (‘ν_Fe-His_‘) was decomposed into component bands with Gaussian functions. The ‘ν_Fe-His_‘ band of rHb(αH87G) at 218 cm^-1^ yielded two component bands at 217 (ν_Fe-His_) and 223 cm^-1^ (ν_Fe-Im_), as illustrated by the dotted red and brown solid lines in Fig 8B, where the sum of the two components bands is shown with a thick broken red line. The ‘ν_Fe-His_‘ band of rHb(βH92G), on the other hand, could not be decomposed into two component bands due to the broadness of the band. However, an assumption of three component bands allowed the band deconvolution as illustrated, i.e. the dotted green, the solid brown and solid blue lines in Fig 8C. The wavenumbers of the three deconvoluted bands are 222 (ν_Fe-His_), 223 (ν_Fe-Im_) and 201 cm^-1^ (ν_Fe-His_). The thick broken green line indicates the sum of the three component bands. The half widths of deconvoluted ν_Fe-His_ components are between 9.0 and 14 cm^-1^, which are smaller than those of ν_Fe-Im_ components (17–22 cm^-1^), as shown in S3 Fig or S1 File.

One mysterious feature is the presence of a weak band at 201 cm^-1^. This may suggest the presence of a small amount of Fe-His heme which has a very low affinity, because in the gel chromatography, rHb(βH92G) was eluted at the tetrameric position with a somewhat broad shape compared with that of Hb A. As a more plausible interpretation, the 201 cm^-1^ band may arise from the conformational heterogeneity of the two α subunits within the T structure [28]. In fact, the resonance Raman spectrum of α(Fe-deoxy)β(Co-deoxy) hybrid Hb excited at 441.6 nm, gave two bands, 201 and 212 cm^-1^, and the two bands were observed only for the T structure [28].

#### Near-UV CD Spectra of Cavity Mutant Hemoglobins

**Fig 9:**
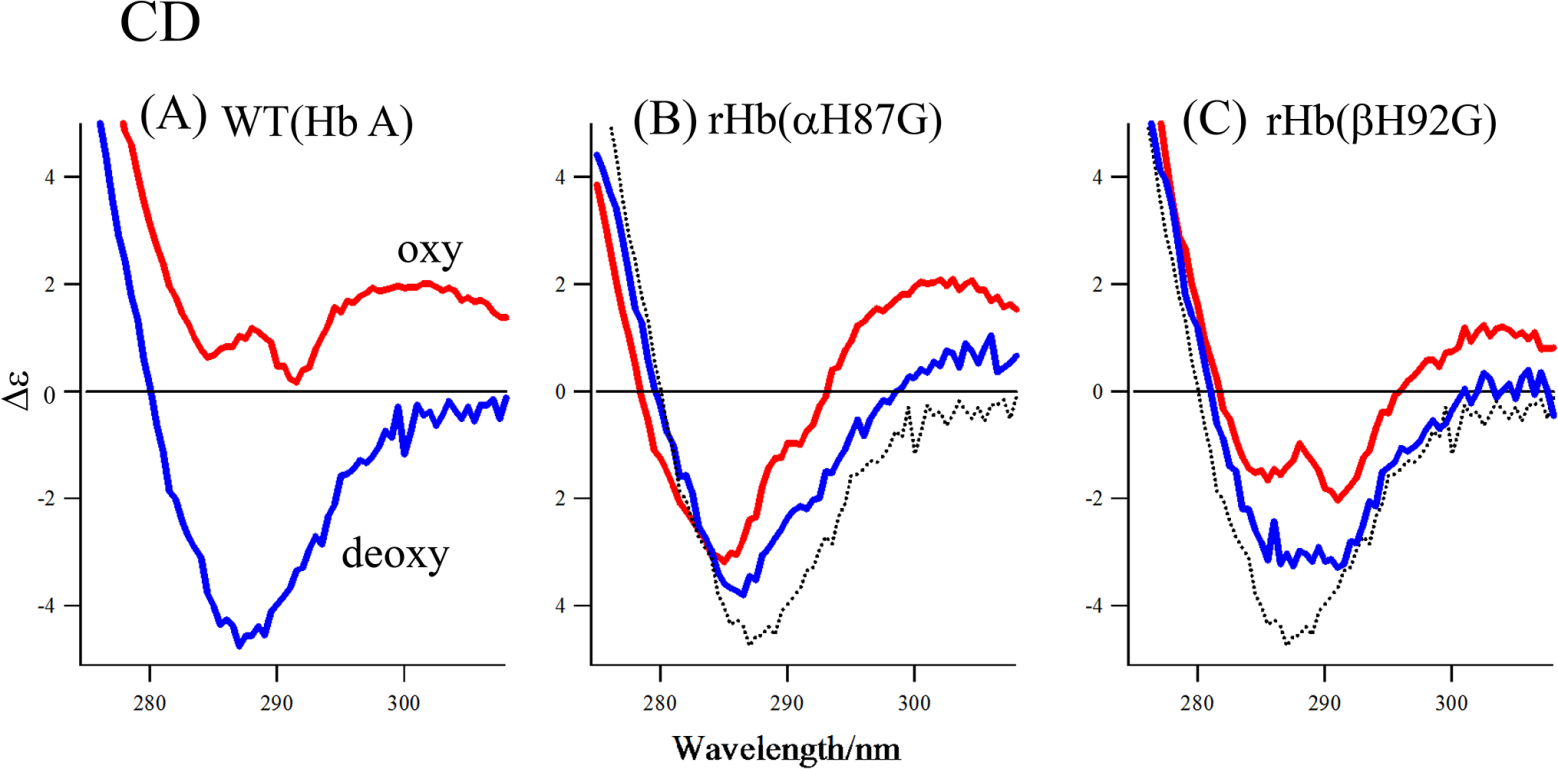
CD spectra of Hb A, rHb(αH87G) and rHb(βH92G). Spectra are Hb A (A), rHb(αH87G) (B) and rHb(βH92G) (C) in the deoxy (blue spectra) and oxy forms (red spectra) in the wavelength region between 275 nm and 320 nm. Hemoglobin concentration, 45 μM (in heme) in a 0.05 M phosphate buffer (pH 7) containing 5 mM imidazole and a metHb reducing system. The broken black lines in (B) and (C) indicate the curve of deoxy Hb A.

Fig 9 shows the near-UV CD spectra of the deoxy- and oxy-forms of Hb A (A), rHb(αH87G) (B) and rHb(βH92G) (C) in the wavelength region from 275 to 310 nm. As shown in Fig 9A, oxyHb A yields small positive CD bands around 287 and 300 nm, while deoxyHb A gives a distinct negative CD band at 287 nm. Both rHb(αH87G) (Fig 9B) and rHb(βH92G) (Fig 9C) yielded a negative CD band in both the oxy- and deoxy-forms, although the negative band in the deoxy form was smaller than that of Hb A, as shown by broken black line. Oxy-rHb(βH92G) exhibits two troughs at 285 and 292 nm, and since the latter is ascribed to the tertiary ‘t’ structure as explained later, it is likely that the quaternary and tertiary structures of oxy-rHb(βH92G) are in part T and t, respectively.

The 287-nm negative CD band of deoxyHb A has been considered to be characteristic of the T quaternary structure [75]. To address its origin, we previously compared the near-UV CD spectra of mutant Hbs, including Hb Rouen (αY140H), rHb(βW37H), rHb(βY145T) and rHb(αY42S), with that of Hb A [76–78] and estimated the relative contributions of the specified aromatic residues through a comparison of the deoxy-minus-oxy difference spectrum. The contribution of Tyrα42 to the negative CD band was small (4%), but Tyrα140 and Tyrβ145 contribution was as large as 30% each [76–78]. The contribution of β37Trp was estimated to be 20%.

**Fig 10:**
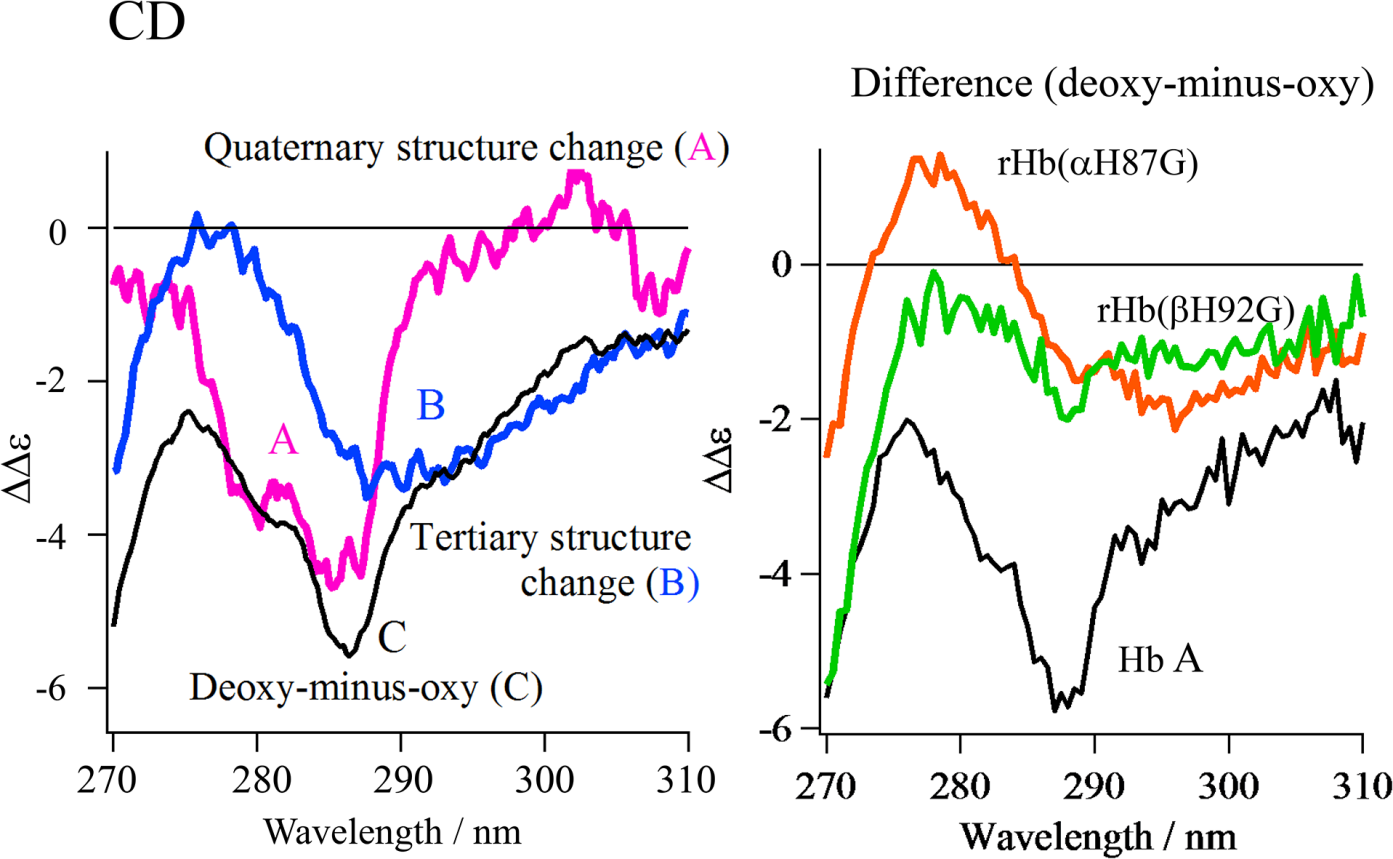
Left: The CD spectral changes due to the quaternary and tertiary structure transition for Hb A. The spectra are the quaternary structure transition (A: pink spectrum) and tertiary structure transition (B: blue spectrum) expected for Hb A and the observed deoxy-minus-oxy difference spectra of Hb A (C: black spectrum). **Right: Comparison of the deoxy-minus-oxy difference spectra of rHb(αH87G) and rHb(βH92G) with that of Hb A.** The difference spectra are Hb A (black spectrum), rHb(αH87G) (orange spectrum) and rHb(βH92G) (green spectrum).

The deoxy-minus-oxy difference CD spectrum of Hb A are illustrated by a black curve in the left panel of Fig 10, where the sum of the contributions from the four aromatic residues mentioned above to the quaternary structure transition is represented by a pink curve (A). The contributions from the tertiary structure changes to the CD spectra upon the change from deoxy (t) to oxy (r) state were estimated from the deoxy-minus-oxy differences of the isolated subunits [79] and superimposed on the same figure with a blue curve (B). It is stressed that a negative CD band is present around 294 nm for the t–r difference, at a longer wavelength than that for the T–R difference. Although the aromatic residues responsible for the negative t–r CD band have not been identified, Trp residues in both chains (Trpα14 and Trpβ15) would be involved.

The right panel in Fig 10 shows the deoxy-minus-oxy difference spectra for rHb(αH87G) and rHb(βH92G) in comparison with Hb A. For rHb(αH87G) (orange), no clear difference band was recognized around 287 nm (at the peak for Hb A), suggesting no quaternary structure change upon O_2_ binding, but the negative t–r difference band is present around 294 nm, indicating that the tertiary structure change only occurred within a frozen T quaternary structure. In the deoxy-minus-oxy difference spectra of rHb(βH92G) (green), on the other hand, a small negative band was observed at 287 nm, suggesting that small quaternary structure changes must have occurred upon O_2_ binding. This change is ascribable to Trpβ37 at the α_1_-β_2_ contact based on the difference between UVRR spectra (E) and (F) in Fig 7. No clear t–r difference band was seen around 294 nm, indicating that no tertiary structure change occurs upon O_2_ binding in rHb(βH92G).

These observations show that in rHb(αH87G), all of the aromatic residues responsible for the near-UV CD (Tyrα42, Tyrα140, Trpβ37, and Tyrβ145) are always in the T quaternary structure and only tertiary structure changes occur upon O_2_ binding/dissociation. In rHb(βH92G), on the other hand, some residues (the penultimate Tyr; Tyrα140 and Tyrβ145) must always be in the T quaternary structure, while some residues (α42Tyr and β37Trp) change from the T to R quaternary structure upon O_2_ binding. Thus, the CD spectral change upon O_2_ binding is simple in rHb(αH87G), in which the quaternary structure changes do not occur at α_1_-β_2_ and *C*-terminal regions and only the tertiary structure change occurs. In contrast, the CD spectral changes of rHb(βH92G) are complicated, because the quaternary structure at the α_1_-β_2_ contact changes upon O_2_ binding, but that at the *C*-terminal region does not, while no tertiary structure changes occur. The quaternary structure changes suggested by the CD spectra for the two cavity mutants are consistent with the results from ^1^H NMR and UVRR studies mentioned above.

## Discussion

### Functional consequences from the lack of an Fe-His bond in α subunits

To investigate the role of the Fe-His bonds of the α- and β-subunits in terms of the functional regulation of oxygen affinity in Hb A, many hybrid Hbs have been studied [25–45,49–51], such as Ni-Fe hybrid Hb, α(Ni)β(Fe^2+^-deoxy) and α(Fe^2+^-deoxy)β(Ni), α-nitrosylβ-deoxy Hb, α(Fe^2+^-NO)β(Fe^2+^-deoxy), and valency hybrid Hb, α(Fe^3+^)β(Fe^2+^-deoxy) and α(Fe^2+^-deoxy)β(Fe^3+^) [35,37,41,52]. Their common shortcoming is that they are unable to take the fully ligand-bound state; in other words, these “tetrameric” hybrid Hbs can bind two O_2_ molecules at most. In contrast, cavity mutant Hbs do bind four O_2_ molecules and accordingly the bound O_2_ –protein interactions in the distal side are fully preserved. The ability of forming the fully O_2_-bound form seems to be indispensable for elucidating possible differences in the role of the Fe-His bonds between the α and β subunits.

The most prominent feature of rHb(αH87G) is that this mutant Hb shows a distinctly biphasic oxygenation curve. This is not due to negative cooperativity and ascribed to the presence of two sets of independent O_2_-binding sites which have widely separated O_2_ affinity values (*P*_50_), 60 mmHg and 3 mmHg. The difference between Hb A and rHb(αH87G) is whether the F-helix is linked to the heme in the α subunits or not. Considering the fact that 1/*K*_1_ can be regarded as *P*_50_ in the deoxy-form of Hb [3], 1/*K*_1_ of Hb A is estimated to be 60 mmHg from Fig 3. The 1/*K*_1_ value obtained for the low O_2_ affinity counterpart of rHb(αH87G) (60 mmHg) is almost the same as that of Hb A. Although 1/*K*_1_ of Hb A is a composite of O_2_ affinities derived from both the α and β subunits, it was reported from the analysis of ^1^H NMR signals that the O_2_ affinities of the α and β subunits of deoxyHb A are alike in the absence of IHP [43,80,81]. Accordingly, it is supposed that the low O_2_ affinity component in the O_2_ equilibrium curve of rHb(αH87G) shown in Fig 2 is assigned to normal β subunits, while its high affinity component is assigned to O_2_ binding to the α subunits with the Fe-Im heme.

Therefore, it is considered that the O_2_ affinity of the β subunits of rHb(αH87G) is maintained low, irrespective of whether O_2_ is bound to the α subunits or not. This is the consequence of the disconnection between the heme and F-helix in the α subunits. Similar phenomena have been observed for α(NO)β(Fe-deoxy) and α(Ni)β(Fe-deoxy) hybrid Hb at low pH, in which the Fe-His and Ni-His bonds are broken in the α subunits [34,53]. In fact, the O_2_ affinity (*P*_50_) of α(NO)β(Fe-deoxy) at pH 5.8 in the presence of IHP, and α(Ni)β(Fe-deoxy) hybrid Hb at pH 6.5 in the presence of IHP, are 65 mmHg and 197 mmHg, respectively [34,53]. In addition, it has been reported that in α(Porphyrin)β(Fe^2+^), which lacks Fe^2+^ in the heme of the α subunits, the *P*_50_ of β heme is 89.3 mmHg at pH 7.4 [32]. In a series of Hbs lacking the Fe-His bond in the α subunits, their Hill constants are between 1 and 1.3 [32,34,53], indicating that their cooperativity is generally little. As these values of *P*_50_ represent the O_2_ affinities of the β subunits, it is suggested that detachment of the heme from the F-helix in the α subunits keeps the O_2_ affinity of the β subunits low, resulting that the tetramer does not switch to R, remaining in T.

In Hb A, the movement of the Fe-His bond following ligand binding to one of the α subunits induces quaternary structure change [4–6], then raises the O_2_ affinity of not only the β subunits but also the other α subunit. In rHb(αH87G), which has the Fe-Im bond in the α subunits with no link to the F-helix, the movement of the Fe-Im bond following ligand binding to the α subunits is not communicated to the F-helix. Therefore, it is supposed that O_2_ affinity of the β subunits remains low.

### Functional consequences from the lack of an Fe-His bond in the β subunits

Although there are many studies [25–45,49–51] on Hbs which lack the Fe-His bond in the α subunits, there are much fewer studies that have been performed for Hbs which lack the Fe-His bond in β subunits. Therefore, it is difficult to estimate the roles of the Fe-His in the β subunits in comparison with the α subunits. We stress that rHb(βH92G) is a rare example which enables an investigation of the Fe-His bond of the β subunits in Hb A. Recombinant-Hb(βH92G) has a high O_2_ affinity, as shown in Figs 3 and 4 (*P*_50_ is 2 mmHg), similar to myoglobin [64]. It has been reported for α(Fe^2+^)β(Porphyrin), having no Fe in the heme of the β subunits, that *P*_50_ is 5.2 mmHg at pH 8.5 and it is non-cooperative (Hill constant, *n* = 1.34) [32]. The *P*_50_ of α(Fe^2+^)β(Porphyrin) at pH 8.5 is similar to that of rHb(βH92G) (= 2 mmHg). Therefore, detachment of the heme from the F-helix in β subunits seems to increase the O_2_ affinity of the α subunits. In other words, the Fe-His bond in the β subunits may be needed in native Hb A to decrease the O_2_ affinity of the α subunits, although it is possible that the detachment only destabilizes the T state shifting the equilibrium toward the R state.

### Structural alteration due to the lack of proximal His in either the α or β subunits

#### Quaternary and tertiary structure changes of the cavity mutants

The ^1^H NMR spectra (Fig 5) show that rHb(αH87G) takes the T structure at the α_1_-β_2_ contact, not only in the deoxy-form but also in the CO-form, while rHb(βH92G) takes the T structure in deoxy-form, but the R structure in the CO-form. The UVRR spectra (Fig 7) are compatible with the results of ^1^H NMR.

The results from NMR (Fig 5) and UVRR (Fig 7) suggest that the quaternary structure change upon CO binding to rHb(αH87G) is much smaller than that of Hb A. Accordingly, the difference peak intensities of the Trp bands in Fig 7E might be attributed to tertiary structural changes. The decrease of Trp intensity in rHb(αH87G) upon ligand binding may correspond to tertiary structure (or state) change from t to r within T quaternary structure. Thus, oxygenation in rHb(αH87G) does not trigger the quaternary structure change from the T to R transition at least and the β subunits remain in T with low affinity, while quaternary structure keeps T structure upon ligand binding to the β subunit as seen in the UVRR and ^1^H NMR spectroscopic data.

On the contrary, rHb(βH92G) which takes the T structure in deoxy-form has higher oxygen affinity and higher ν_Fe-His_ frequency (222 cm^-1^) than those of deoxyHb A. This may cause tertiary structure (or state) change from t to r within T quaternary structure due to the lack of proximal His in β subunit. A tertiary structure (or state) change from t to r in this case may be attributable to shortening of Fe-His bond in α subunit.

**Fig 11:**
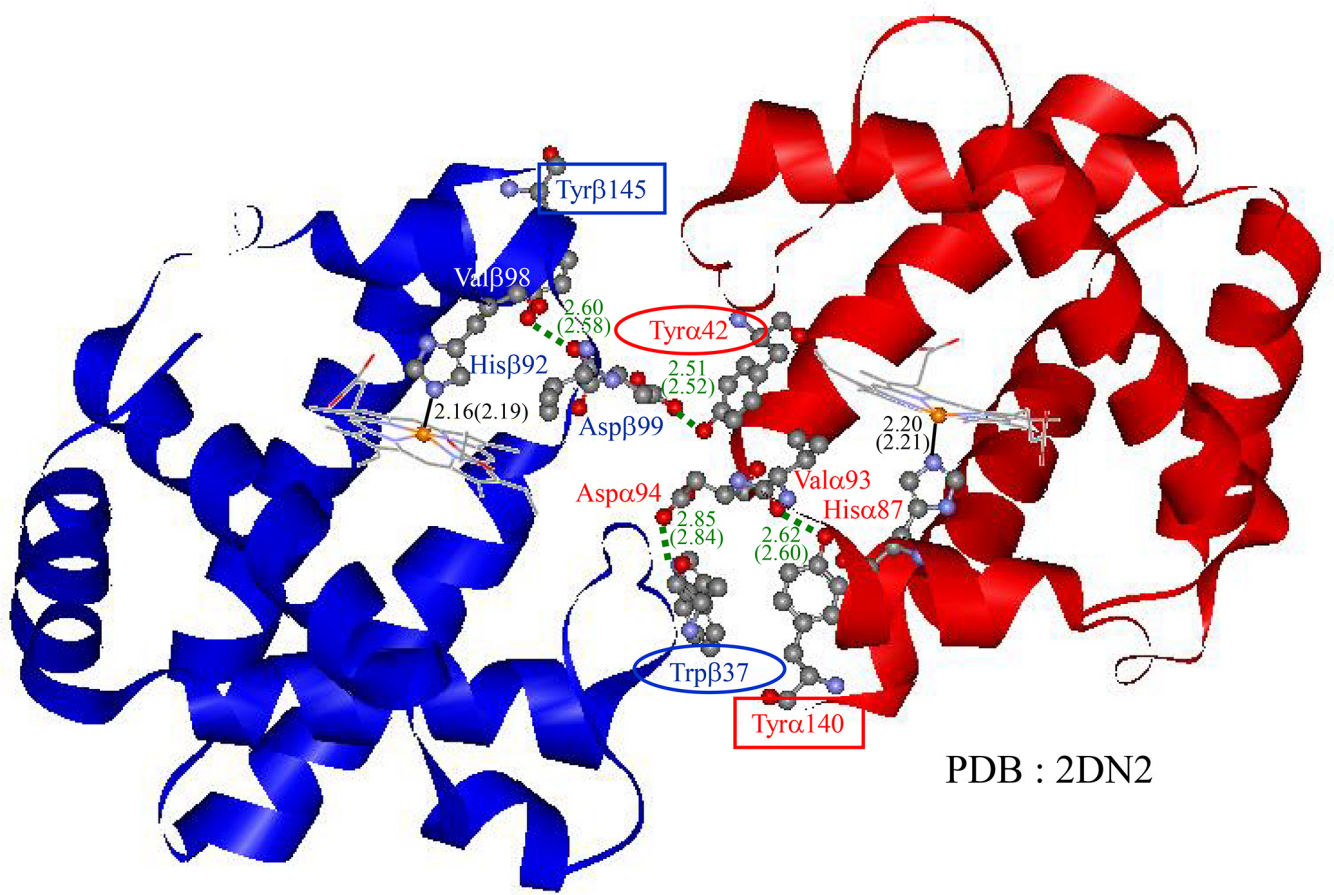
Intersubunit interactions of deoxy Hb A. Intersubunit interactions at the interface between the α_1_ and β_2_ subunits and *C*-terminal region of deoxy Hb A were revealed by X-ray crystallography [46]. Tyrα42 is hydrogen bonded with Aspβ99, and Trpβ37 is hydrogen bonded with Aspα94 of G-helix in the inter-subunit surfaces, respectively. Tyrα140 in H-helix is interacting with a residue of another subunit and is also hydrogen bonded with Valα93 as the intra-subunit interaction. Tyrβ145 in H-helix is hydrogen bonded with Valβ98 as an intra-subunit interaction. The F-helix is connected to the proximal His, while the E-helix is associated with an external ligand on the distal side. Tyrα140 and Tyrβ145 are contained in the *C*-terminal region.

The near-UV CD spectra of Hb A provide interesting information on the tertiary and quaternary structure changes that occur upon O_2_ binding. The CD bands of deoxyHb A consist of two components, one at 287 nm which mainly reflects the quaternary structure transition and another around 294 nm that mainly reflects tertiary structure changes. The quaternary structure transition of Hb A upon O_2_ binding involves structural changes in the aromatic residues in the α_1_-β_2_ contact (Tyrα42 and Trpβ37) and *C*-terminal regions (Tyrα140 and Tyrβ145) (Fig 11) [4–6,46]. The former changes were detected by ^1^H NMR and the latter by near-UV CD, while all can be detected by UVRR. It was clarified that 70% of CD band intensity around 287 nm of deoxyHb A reflects the *C*-terminal region (Tyrα140 and Tyrβ145) [76–78].

The CD bands of rHb(αH87G) showed a distinct negative CD band at 287 nm in both the deoxy- and oxy-forms, indicating that oxy-rHb(αH87G) as well as the deoxy one takes a T quaternary structure in both the α_1_-β_2_ contact and *C*-terminal regions. In the deoxy-minus-oxy difference CD spectrum shown in Fig 10 (right-orange), the band shape around 294 nm is negative, like that of Hb A, indicating the occurrence of the t to r tertiary structure changes upon O_2_ binding similar to Hb A. On the other hand, rHb(βH92G) gave two CD bands at 285 and 292 nm in both the deoxy- and oxy-forms (Fig 9C). Since the 287-nm band was 30% smaller than that of deoxyHb A, it seemed that the *C*-terminal region mostly keeps the T structure and the 30% would have arisen from the T to R structural changes in the α_1_-β_2_ contact region. Tertiary structure changes did not occur to rHb(βH92G), as judged from the CD spectral change around 292 nm upon O_2_ binding. This indeed gave no trough at 294 nm in Fig 10 (right, green). Thus the near-UV CD spectra of Hb A, rHb(αH87G) and rHb(βH92G) upon ligand binding may give information about tertiary structure changes.

In the decomposed spectra of Fig 8, the ν_Fe-His_ bands for the normal subunits were observed at 217 and 222 cm^-1^ for deoxy-rHb(αH87G) and deoxy-rHb(βH92G), respectively. It has been established that the ‘ν_Fe-His_’ frequency reflects the O_2_ affinity for Hb A [51]; its 6 cm^-1^ shift to higher frequencies corresponds to a 100 times higher affinity. Thus, the higher ν_Fe-His_ frequency of deoxy-rHb(βH92G) compared with deoxy-rHb(αH87G) is consistent with its higher O_2_ affinity.

In a rHb(βH92G) of deoxy-form, UVRR and ^1^H NMR show T quaternary structure. Based on the TTS model, T quaternary structure of rHb(βH92G) contains more r tertiary state than Hb A does it within T quaternary structure, under the assumption that both r and t states of this mutated Hb are the same as those of Hb A. This means that detachment of the heme from the F-helix in β subunits causes an effect that increases r tertiary state within T quaternary structure. Therefore, oxygen affinity in a rHb(βH92G) of deoxy-form may increase than that in deoxyHb A. In previous paragraph, we described that tertiary structure changes had not occurred upon O_2_ binding to rHb(βH92G) from the CD results (Fig 10). It is inferred that quaternary structure change occurred and that tertiary structure changes did not occur upon O_2_ binding to rHb(βH92G), as deoxy-rHb(βH92G) has as r state as oxy-rHb(βH92G). However, it is noted that tertiary structures of rHb(βH92G) observed with CD spectra of both deoxy and oxy-forms remain in t tertiary structure, although t tertiary structure observed with CD spectra does not always mean t state. Considering an oxygen affinity curve of Fig 3, detachment of the heme from the F-helix in the β subunits of cavity mutant Hb, rHb(βH92G), can cause increase of the O_2_ affinity of the α subunits in the deoxy forms.

Furthermore, as previously mentioned, this means that based on the TTS model, the r tertiary state of rHb(βH92G) within T quaternary structure is almost the same as r tertiary state of swMb which has no quaternary structure. This might suggest that detachment of the heme from the F-helix in β subunits in rHb(βH92G) promotes a high oxygen affinity of α subunit similar to that of swMb. Comparison of oxygen affinity of rHb(βH92G) with that of swMb seems to demonstrate that existence of Fe-His bond in β subunit is indispensible for a decrease of the oxygen affinity in α subunit of native tetramer Hb A, because we consider from UVRR and ^1^H NMR results that deoxy form of rHb(βH92G) maintains a tetramer structure.

As shown in Figs 3 and 4, a rHb(βH92G) at high saturation (= upon full oxygen bindings) exhibits the same oxygen affinity as Hb A. Based on the TTS model, rHb(βH92G) of ligand-bound form stays in the same R state (R quaternary structure) with r tertiary structure as that of Hb A, indicating that the intrinsic functional properties of the tertiary state, r of a rHb(βH92G) are the same as those of Hb A.

#### Role of the Fe-His bond in cooperative O2 binding

The present study of the cavity mutant Hbs rHb(αH87G) and rHb(βH92G) demonstrates the functional and structural roles of the proximal His in the α and β subunits in cooperative oxygen binding. Cooperativity disappeared when the Fe-His bond in either of the α or β subunits was lost. Detachment of the heme from the F-helix in the α subunits maintains quaternary structure of rHb(αH87G) in T for both the α_1_-β_2_ contact- and *C*-terminal regions, even after ligand binding to not only α(Fe-Im) but also β(Fe-His). Namely the tetramer is frozen in the T quaternary structure. In contrast, the detachment of heme from the F-helix in the β subunits in rHb(βH92G) maintains its quaternary structure in T at both the α_1_-β_2_ contact- and the *C*-terminal regions in the deoxy-form, but ceased to maintain it in the α_1_-β_2_ contact region upon O_2_ (ligand) binding, making the O_2_ affinity of the remaining subunits higher than that in native Hb A.

In native Hb, movement of the Fe-His in the α subunit upon ligand binding induces quaternary structure change through the inter-subunit hydrogen bonding network between Tyrα42 and Aspβ99 and between Aspα94 and Trpβ37 at the α_1_-β_2_ contact region [4–6,46,65], which raises the O_2_ affinity of the β subunits. Quaternary structure change upon ligand binding in rHb(βH92G), however, maintains the T quaternary structure of the *C*-terminal region. Thus, the quaternary structure at the *C*-terminal region does not directly correlate with the magnitude of strain exerted on the α(Fe-His) and β(Fe-His) bonds.

### Relation between the higher order structures and oxygen affinity in Hb A

The spectroscopic features of the higher order structures obtained from the present experiments are summarized in Table 2.

**Table 2:** Summary of conformations of the cavity mutant Hbs and oxygen binding properties of the normal subunits.

	rHb(αH87G)		rHb(βH92G)	
	Deoxy	Liganded	Deoxy	Liganded
Quaternary structures				
α_1_-β_2_ contact regions^a^	**T**	**T**	**T**	**R**
C-terminal regions^b^	**T**	**T**	**T**	**T**
Tertiary structure^c^	**t**	**r**	**t**	**t**
Properties of normal subunits^d^				
ν_Fe-His_	217 cm^-1^		222 cm^-1^	
O_2_ affinity	verylow (**T**)	verylow (**T**)	high **(R**)	high **(R**)

^a^, ^1^H NMR and UVRR;

^b^, UVRR and near-UV CD (287 nm);

^c^, near UV CD (294 nm);

^d^, visible RR and OEC.

#### α_1_-β_2_ contact region

Recombinant Hb(αH87G) takes the T quaternary structure at the α_1_-β_2_ region (Tyrα42 and Trpβ37) in both the deoxy- and liganded forms, as shown by the ^1^H NMR and UVRR spectra. In contrast, rHb(βH92G) takes the T structure in the deoxy-form, but changes into the R structure upon ligand binding. These results indicate that ligand binding to α(Fe-His) heme causes the changes in the α_1_-β_2_ contact region.

#### *C*-terminal region

In the deoxy-minus-CO difference spectra of UVRR, changes in the Tyr RR bands of rHb(βH92G) were half those in Hb A. We previously showed that changes in the Tyr RR bands were attributable to Tyrα42 [82] and Tyrα140 [83]. As the change in Tyrα42 upon CO binding was demonstrated by ^1^H NMR in Fig 5, it is suggested that Tyrα140 retains the T structure in rHb(βH92G) even after CO binding. The negative CD band at 287 nm in deoxyHb A (T structure marker band) is composed of contributions from four residues (Tyrα42, Tyrα140, Tyrβ145 and Trpβ37) (Fig 11), and among them the contributions of Tyrα140 and Tyrβ145 (*C*-terminal region) are larger than those of Tyrα42 and Trpβ37 (α_1_-β_2_ contact) [76–78]. Although the negative CD band of oxy-rHb(βH92G) is smaller than that of rHb(αH87G), both cavity mutant Hbs assume a T-quaternary structure at the *C*-terminal region.

In Table 2, we summarize the quaternary structure (UVRR, ^1^H NMR and the negative CD band at 287 nm) and the tertiary structure determined on the basis of CD pattern around 294 nm. The ν_Fe-His_ frequency appears to reflect the total degree of strain exerted on the Fe-His bond by the higher order protein structures. These results consistently indicate that rHb(αH87G) takes the T quaternary structure in both the deoxy- and liganded forms, and that rHb(βH92G) takes the T quaternary structure in the deoxy-form, but upon ligand bindings rHb(βH92G) changes to the R structure at the α_1_-β_2_ contact region, while remaining in the T structure at the *C*-terminal region. It is really puzzling for the liganded rHb(βH92G) that part of the structure is assigned to the quaternary T and part to the R quaternary conformation. It might be more understandable when we are referring this result to TTS model. On the basis of this model, rHb(βH92G) is a novel conformation that is in one part t and in another part is r. Even if we accept TTS model for the present results, the different roles of the α and β subunits in the α_2_β_2_ tetramer for cooperative oxygen binding could not be interpreted by the model. This is a new finding for understanding cooperativity of Hb A. Therefore, we will especially stress significance to discriminate α with β subunit on elucidating cooperativity of Hb A.

### Regulation mechanism of the O_2_ affinity of Hb A (the communication pathway between α and β subunits)

**Fig 12:**
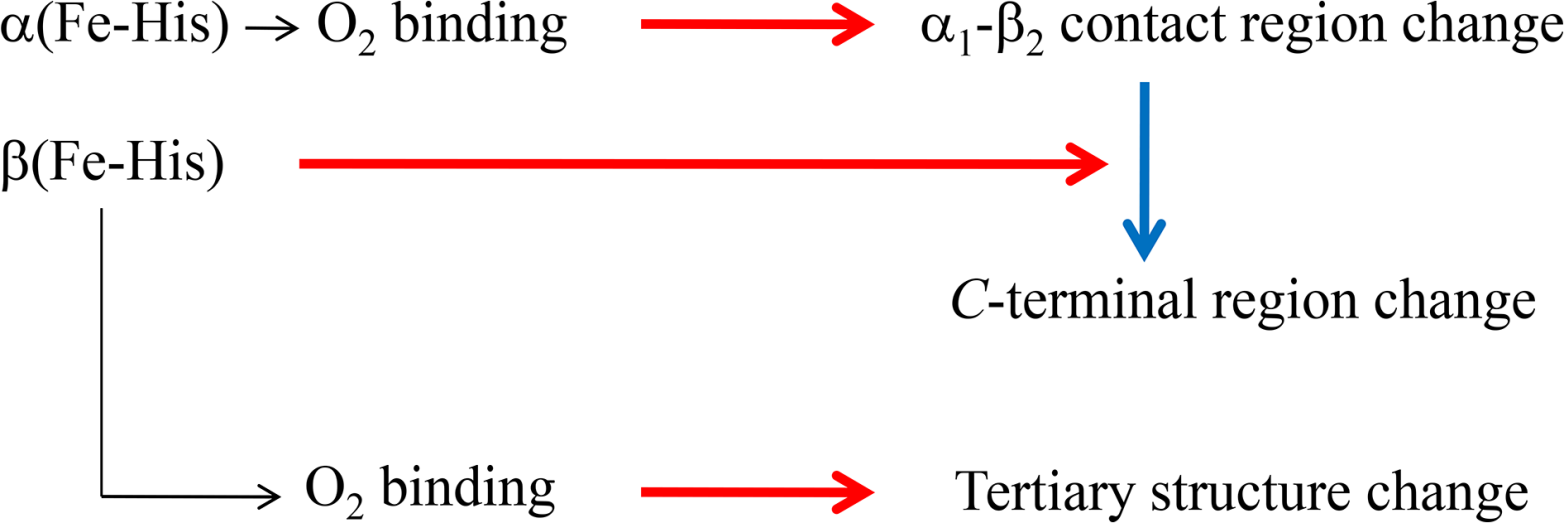
Possible roles of the Fe-His bonds of α and β subunits on cooperative O_2_ binding.

The present observations suggest that communication between α and β subunits of Hb upon ligand binding takes place, as shown in Fig 12. The α_1_-β_2_ contact changes are induced by ligand binding to α(Fe-His), first in Hb A, and subsequently the *C*-terminal regions are changed by the ligand binding to β(Fe-His) or the movement of the F-helix of β subunits *via* changes that occur in the α_1_-β_2_ contact regions upon ligand binding to α(Fe-His). In contrast, ligand binding to β(Fe-His), if it occurred first in Hb A, does not induce a quaternary structure change in either the *C*-terminal regions or α_1_-β_2_ contact, but induces tertiary structure change.

In the absence of an Fe-His bond in the α subunit for rHb(αH87G), the ligand binding to the α subunit does not change the hydrogen bonding network at the α_1_-β_2_ contact, and as a result the β subunit is retained in the T structure. In contrast, detachment of heme from the F-helix in the β subunits makes the O_2_ affinity of the α subunits higher, and in addition, tends to dissociate the Hb tetramer into two dimers [54]. This implies that the Fe-His bond of β subunit in the native HbA plays an important role in decreasing the O_2_ affinity of the α subunits. Thus, both the α and β subunits regulate the O_2_ affinity of the other subunit. Consequently, the regulatory mechanism of O_2_ affinity and a communication route from the α to β subunits are different than from the β to α subunits. In other words, the α subunits control the O_2_ affinity of the β subunits through the quaternary structure change that occurs concomitantly with ligand binding to the α subunits, but the β subunits control the O_2_ affinity of the α subunits by change in the *C*-terminal region through movement of the β(Fe-His) bond.

In conclusion, the present study shows that the Fe-His bond in the β subunits decreases the O_2_ affinity of the α subunits in native Hb A, and its relaxation by successive O_2_ binding is critical for cooperative O_2_ binding. The Fe-His bond of the α subunits in native Hb A, in contrast, is essential for increasing the O_2_ affinity of the β subunits so as to yield a large *K*_4_ value. We believe that spectroscopic data of cavity mutant Hbs observed in this study may give more concrete information about t and r states by discriminating α from β subunit based on the TTS model, although differences between t and r states of Hb A and t and r states of cavity mutant Hb, rHb(αH87G) and rHb(βH92G) need to be sufficiently considered.

## Supporting Information

S1 Fig 600 MHz ^1^H NMR spectra of rHb(αH87G) and rHb(βH92G). Upper and lower spectra are deoxy- and CO rHb(αH87G) (left), and deoxy- and COrHb(βH92G) (right) between 10 and 30 ppm at pH 7.0 and 25 °C, respectively. The hemoglobin concentrations of rHb(αH87G) and rHb(βH92G) were 800 and 500 μM, respectively, on a heme basis in 0.05 M phosphate buffer (pH 7.0). In addition, rHb(αH87G) and rHb(βH92G) contained 10 mM imidazole.
(TIF)

S2 Fig In the 229-nm excited UVRR spectrum and the difference spectra (Hb A-minus-cavity mutant Hbs). Hb A–rHb(βH92G) (deoxy) (A), Hb A–rHb(αH87G) (deoxy) (B), Hb A–rHb(βH92G) (CO) (C), Hb A–rHb(αH87G) (CO) (D), Hb A–rHb(βH92G) (deoxy) (E), Hb A–rHb(βH92G) (CO) (F). A spectrum of imidazole (10 mM) is shown by green line. The difference spectra of (E) and (F) are the difference spectra calculated from ((A)–imidazole) and from ((C)–imidazole), respectively. The hemoglobin concentration was 200 μM (in heme) in a 0.05 M phosphate buffer (pH 7.0) containing 0.2 M SO_4_^2-^ as the internal intensity standard. In addition, rHb(αH87G) and rHb(βH92G) contained 10 mM imidazole. The difference spectra were obtained so that the Raman band of SO_4_^2-^ (980 cm^-1^) could be abolished. The spectra shown are an average of 13 scans.
(TIF)

S3 Fig The 441.6-nm excited visible RR spectra of deoxyHb A (A), deoxy-rHb(αH87G) (B) and deoxy-rHb(βH92G) (C). The hemoglobin concentration was 200 μM (in heme) in a 0.05 M phosphate buffer, pH 7.0. In addition, rHb(αH87G) and rHb(βH92G) contained 10 mM imidazole. For rHb(αH87G), deconvoluted components, ν_Fe-His_ and a ν_Fe-Im_ are indicated by a thin dotted red line and a brown solid line, respectively. For rHb(βH92G), deconvoluted components, a ν_Fe-His_ (high), a ν_Fe-His_ (low) and a ν_Fe-Im_ are indicated by a thin dotted green, a solid blue line and a solid brown line, respectively. Fitted parameters are shown on the right hand of each spectrum.
(TIF)

S4 Fig UV-Vis absorption spectra of CO-rHb(αH87G) containing 1mM (solid) and 6 mM imidazole (dotted), respectively. Buffer solutions are 0.05 M phosphate buffer at pH 7.0.
(TIF)

S5 Fig UV-Vis absorption spectra of CO-rHb(βH92G) containing 1 mM (solid) and 6 mM imidazole (dotted), respectively. Buffer solutions are 0.05 M phosphate buffer at pH 7.0.
(TIF)

S6 Fig Hill plots of oxygen equilibrium curve of rHb(αH87G). Solution conditions are at pH 7.4, black closed circle (**●**), at pH 7.9, open circle (**○**), and at pH 7.4 in the presence of IHP, asterisk (*).
(TIF)

S7 Fig Hill plots of oxygen equilibrium curve of rHb(βH92G). Solution conditions are at pH 7.4, black closed circle (●), at pH 7.9, open circle (○), and at pH 7.4 in the presence of IHP, asterisk (*).
(TIF)

S1 File Figures from S1 Fig to S7 Fig are contained. (PPT)
