# Supplementary figures and images for: An Origin of Cooperative Oxygen Binding of Human Adult Hemoglobin: Different Roles of the α and β Subunits in the α2β2 Tetramer
Source: PLoS One. 2015 Aug 5;10(8):e0135080. doi: 10.1371/journal.pone.0135080 (PMC4526547; doi:10.1371/journal.pone.0135080)

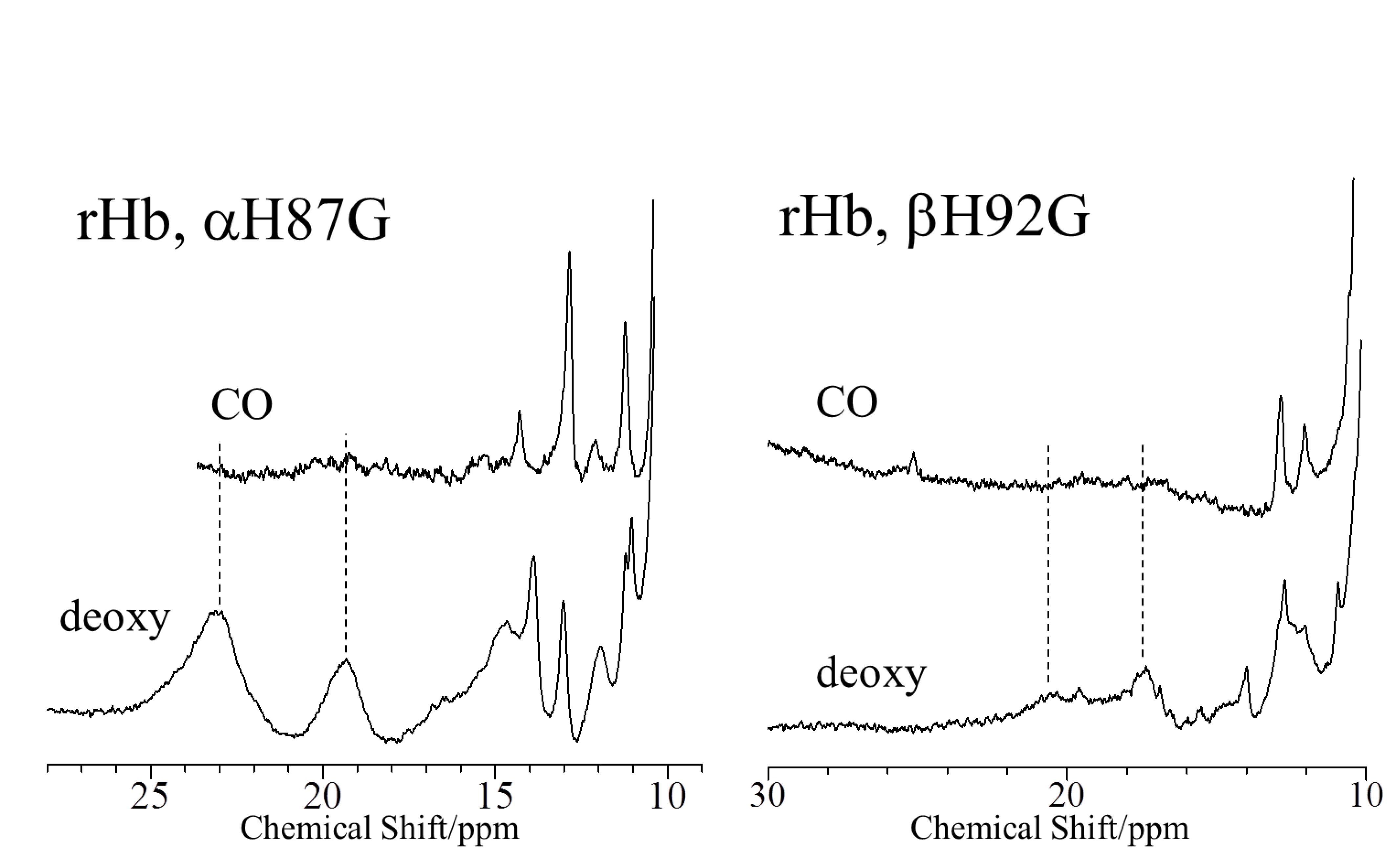

Supplement: S1 Fig — Upper and lower spectra are deoxy- and CO rHb(αH87G) (left), and deoxy- and COrHb(βH92G) (right) between 10 and 30 ppm at pH 7.0 and 25 °C, respectively. The hemoglobin concentrations of rHb(αH87G) and rHb(βH92G) were 800 and 500 μM, respectively, on a heme basis in 0.05 M phosphate buffer (pH 7.0). In addition, rHb(αH87G) and rHb(βH92G) contained 10 mM imidazole. (TIF) [file pone.0135080.s001.tif]

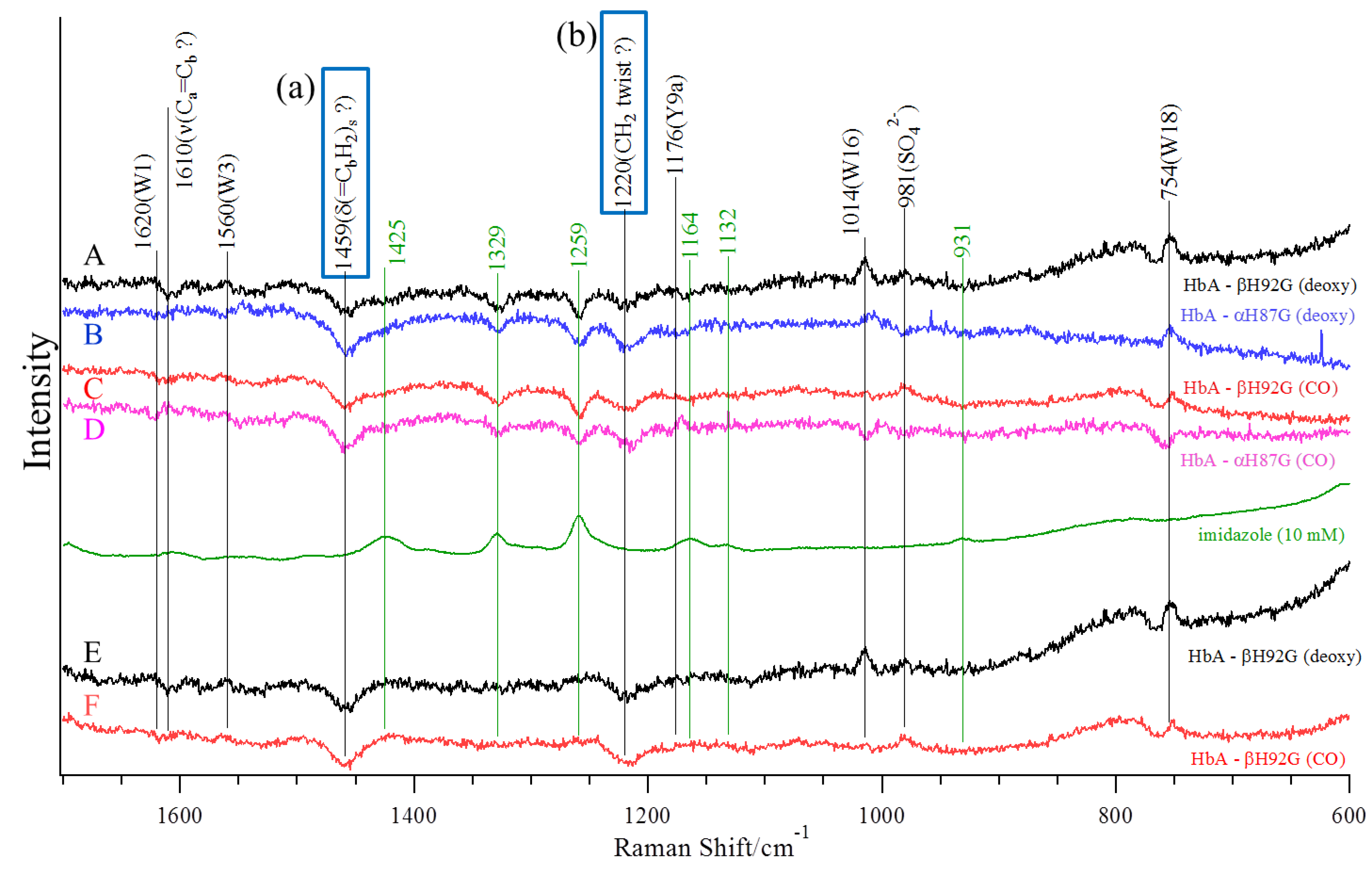

Supplement: S2 Fig — Hb A–rHb(βH92G) (deoxy) (A), Hb A–rHb(αH87G) (deoxy) (B), Hb A–rHb(βH92G) (CO) (C), Hb A–rHb(αH87G) (CO) (D), Hb A–rHb(βH92G) (deoxy) (E), Hb A–rHb(βH92G) (CO) (F). A spectrum of imidazole (10 mM) is shown by green line. The difference spectra of (E) and (F) are the difference spectra calculated from ((A)–imidazole) and from ((C)–imidazole), respectively. The hemoglobin concentration was 200 μM (in heme) in a 0.05 M phosphate buffer (pH 7.0) containing 0.2 M SO4 2- as the internal intensity standard. In addition, rHb(αH87G) and rHb(βH92G) contained 10 mM imidazole. The difference spectra were obtained so that the Raman band of SO4 2- (980 cm-1) could be abolished. The spectra shown are an average of 13 scans. (TIF) [file pone.0135080.s002.tif]

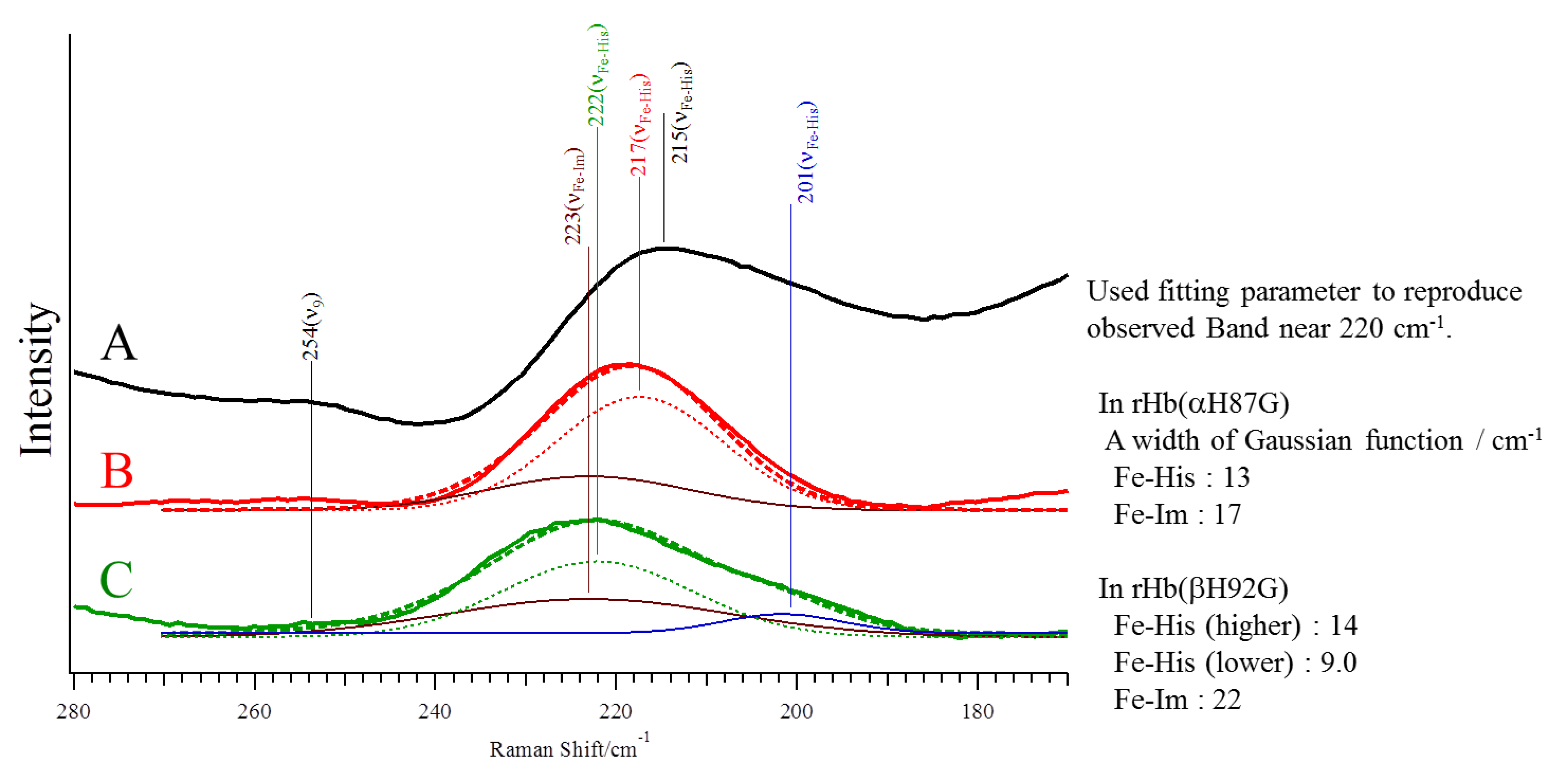

Supplement: S3 Fig — The hemoglobin concentration was 200 μM (in heme) in a 0.05 M phosphate buffer, pH 7.0. In addition, rHb(αH87G) and rHb(βH92G) contained 10 mM imidazole. For rHb(αH87G), deconvoluted components, νFe-His and a νFe-Im are indicated by a thin dotted red line and a brown solid line, respectively. For rHb(βH92G), deconvoluted components, a νFe-His (high), a νFe-His (low) and a νFe-Im are indicated by a thin dotted green, a solid blue line and a solid brown line, respectively. Fitted parameters are shown on the right hand of each spectrum. (TIF) [file pone.0135080.s003.tif]

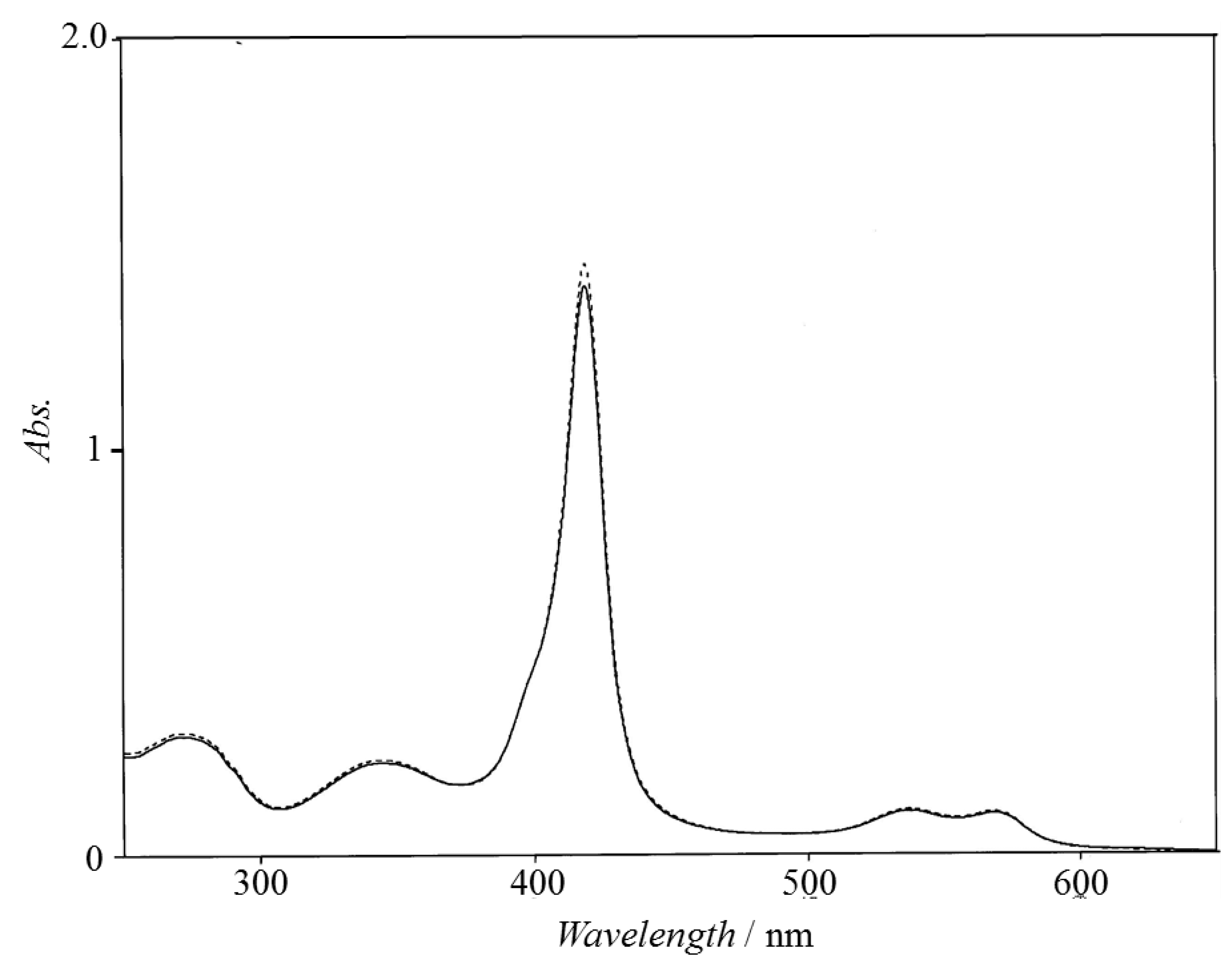

Supplement: S4 Fig — Buffer solutions are 0.05 M phosphate buffer at pH 7.0. (TIF) [file pone.0135080.s004.tif]

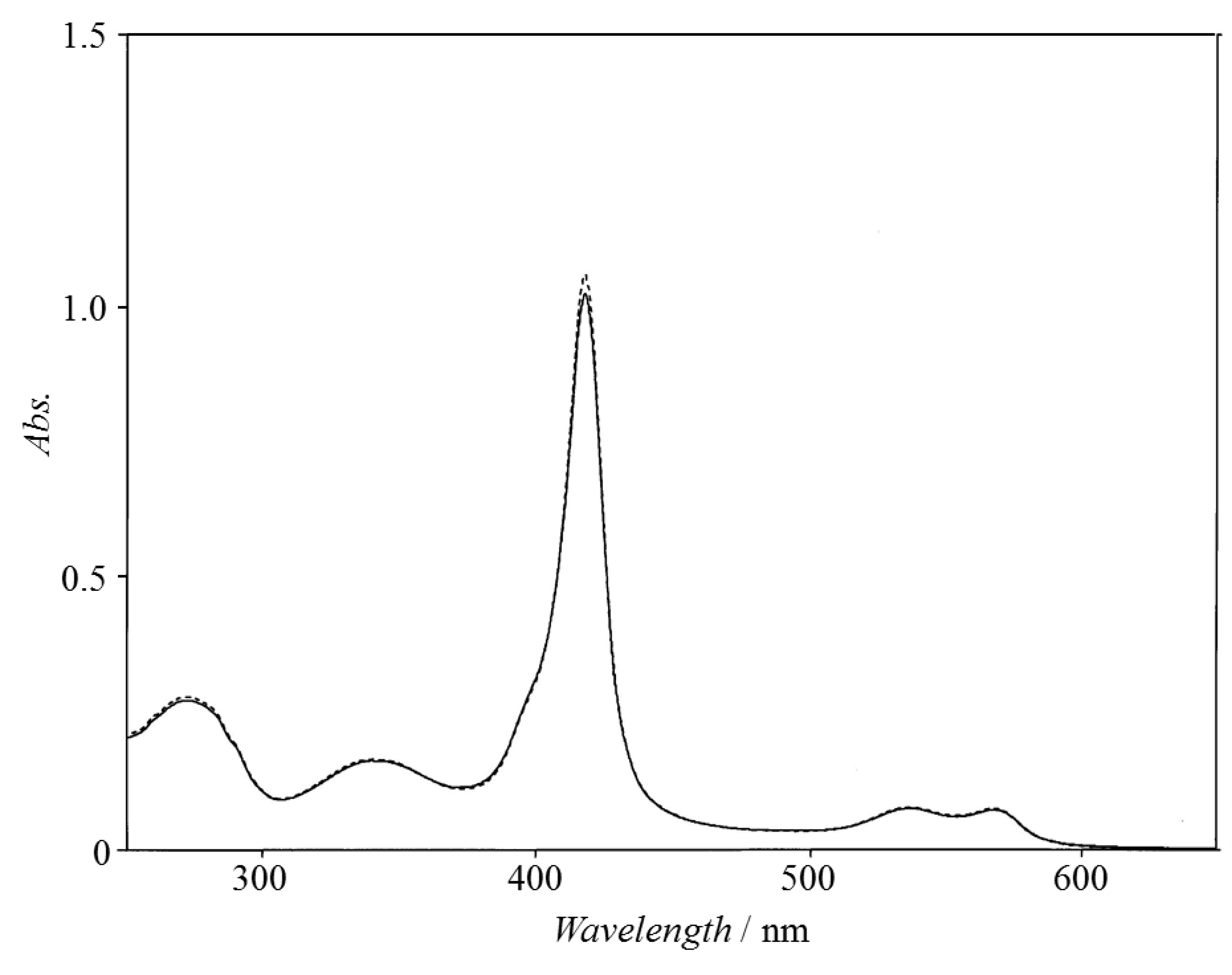

Supplement: S5 Fig — Buffer solutions are 0.05 M phosphate buffer at pH 7.0. (TIF) [file pone.0135080.s005.tif]

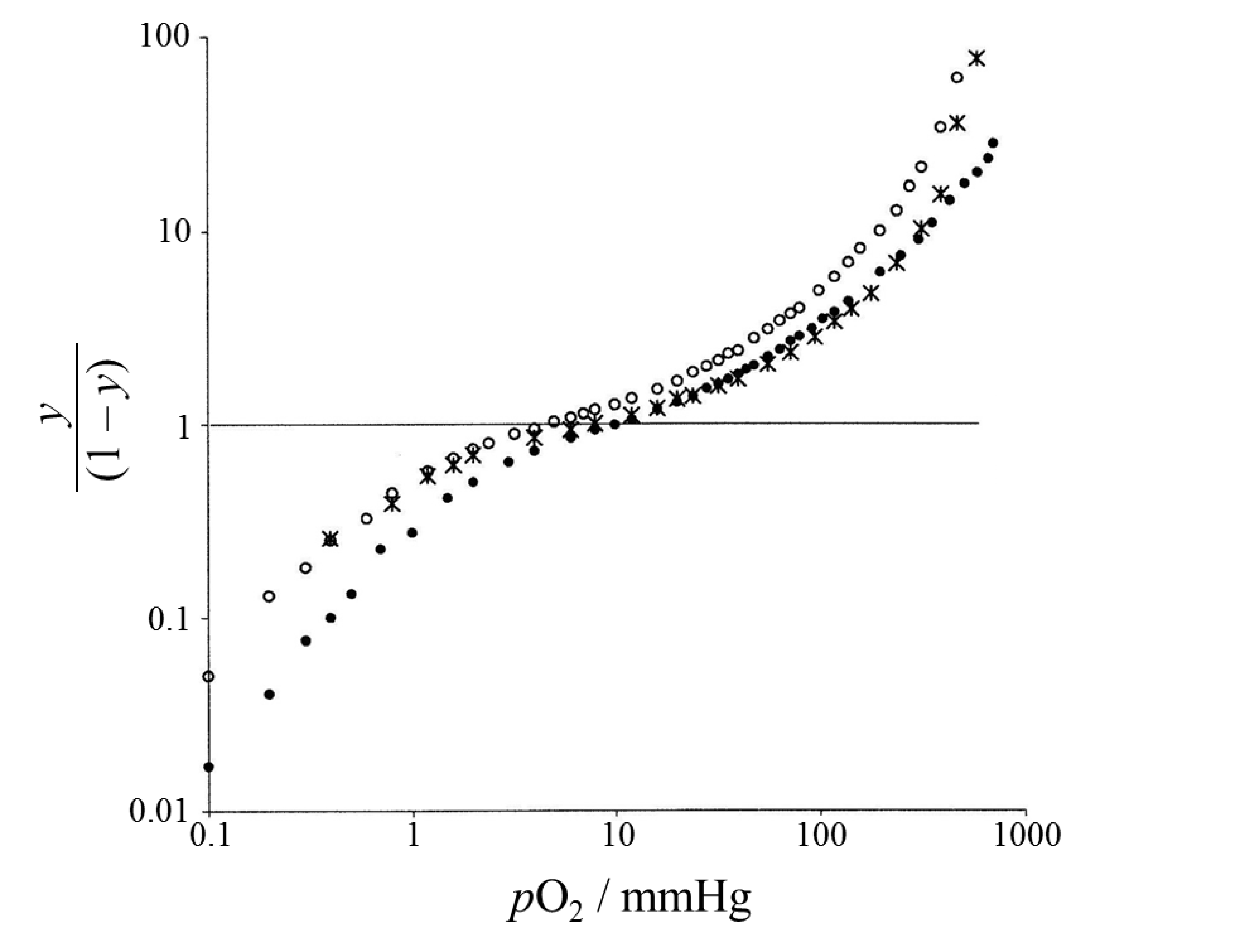

Supplement: S6 Fig — Solution conditions are at pH 7.4, black closed circle (●), at pH 7.9, open circle (○), and at pH 7.4 in the presence of IHP, asterisk (*). (TIF) [file pone.0135080.s006.tif]

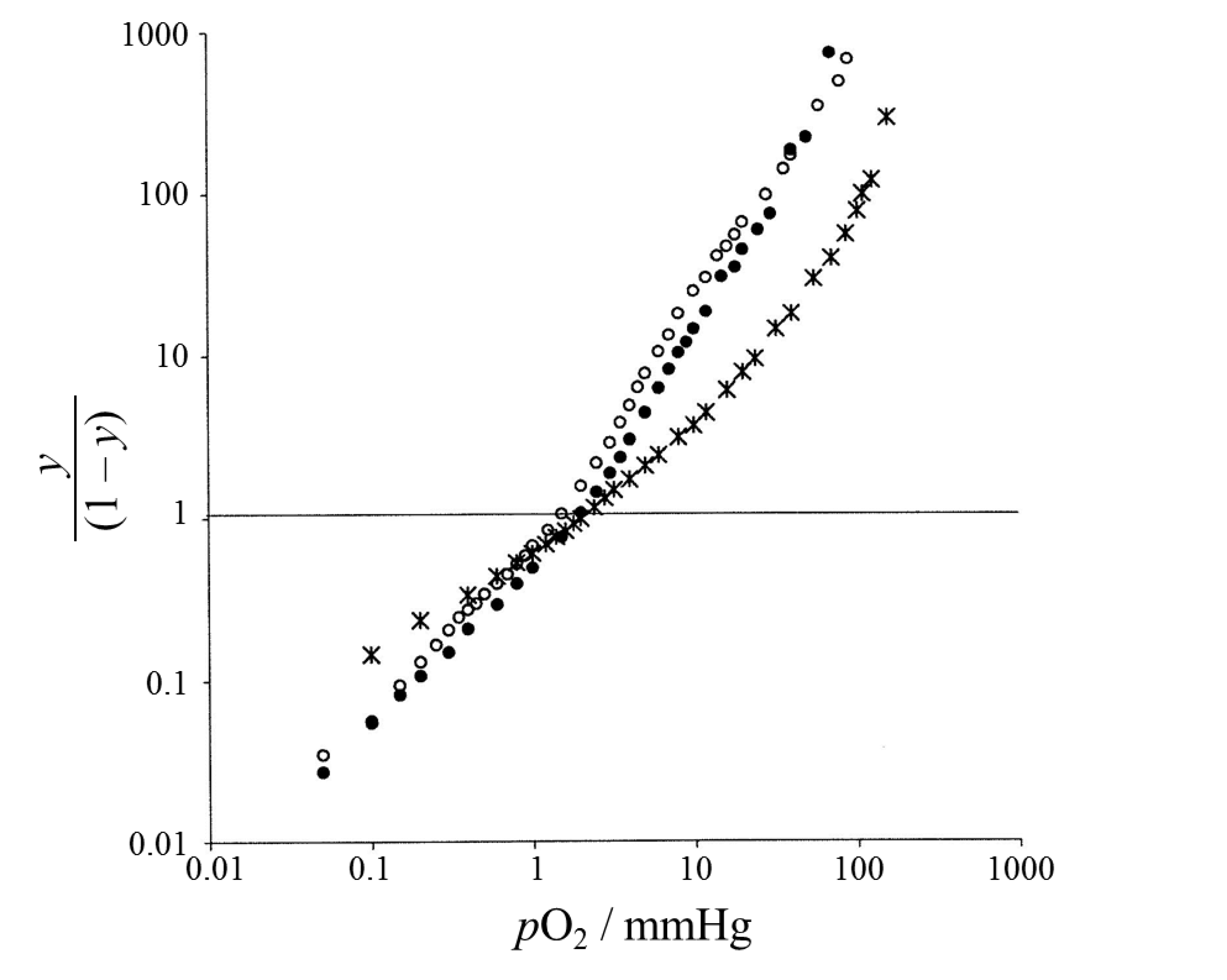

Supplement: S7 Fig — Solution conditions are at pH 7.4, black closed circle (●), at pH 7.9, open circle (○), and at pH 7.4 in the presence of IHP, asterisk (*). (TIF) [file pone.0135080.s007.tif]

## Slide 1
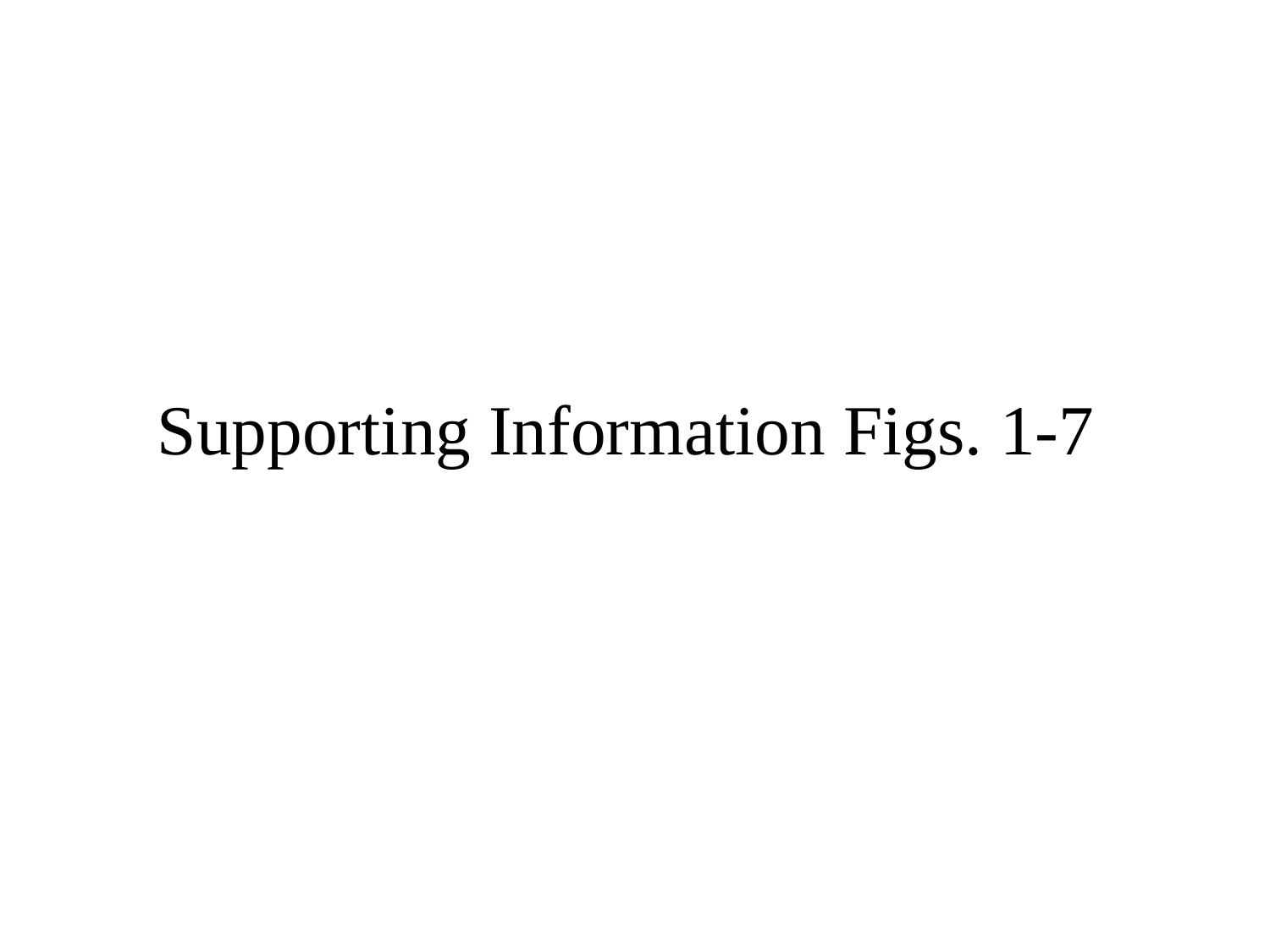

Supporting Information Figs. 1-7

## Slide 2
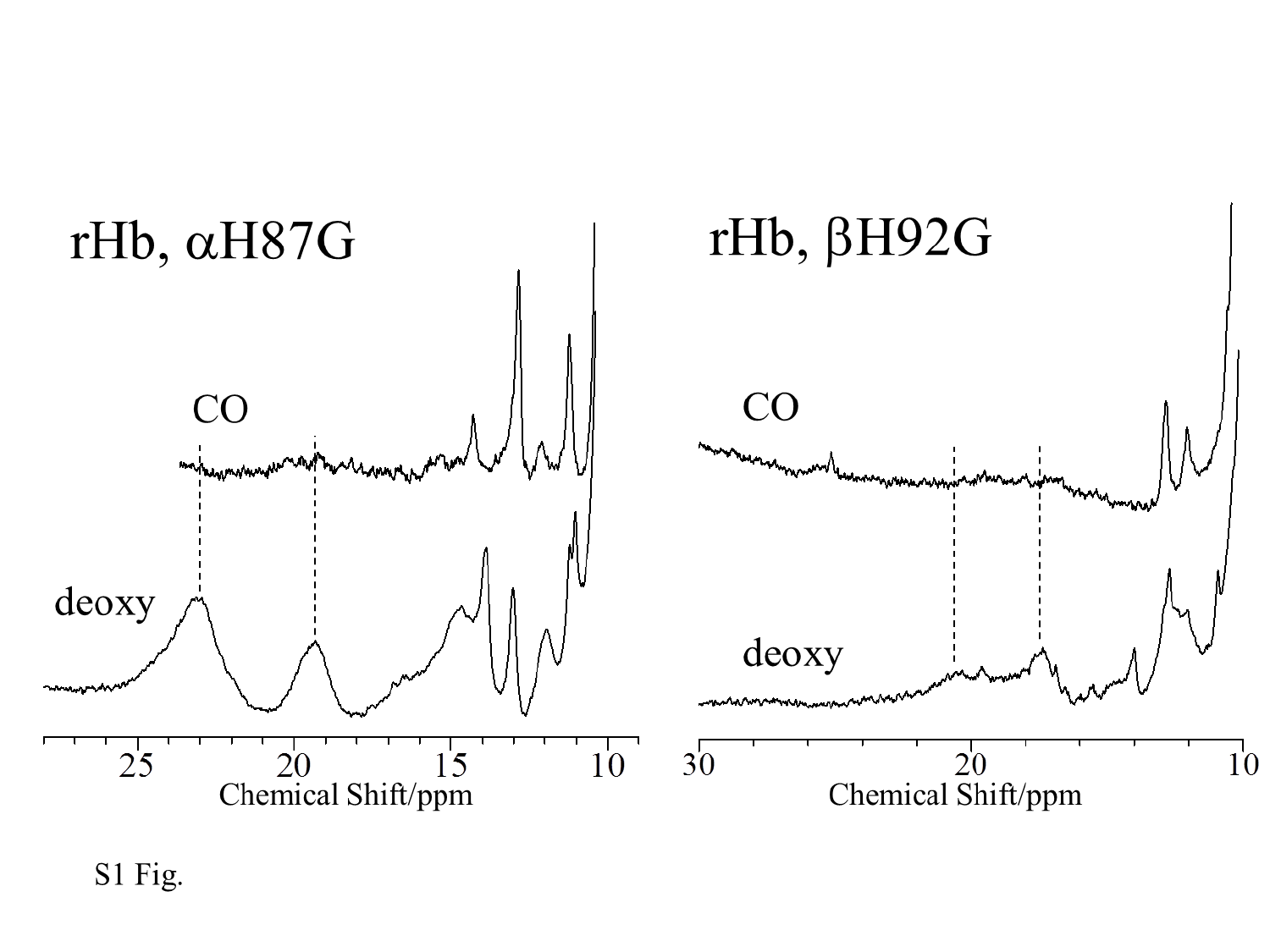

## Slide 3
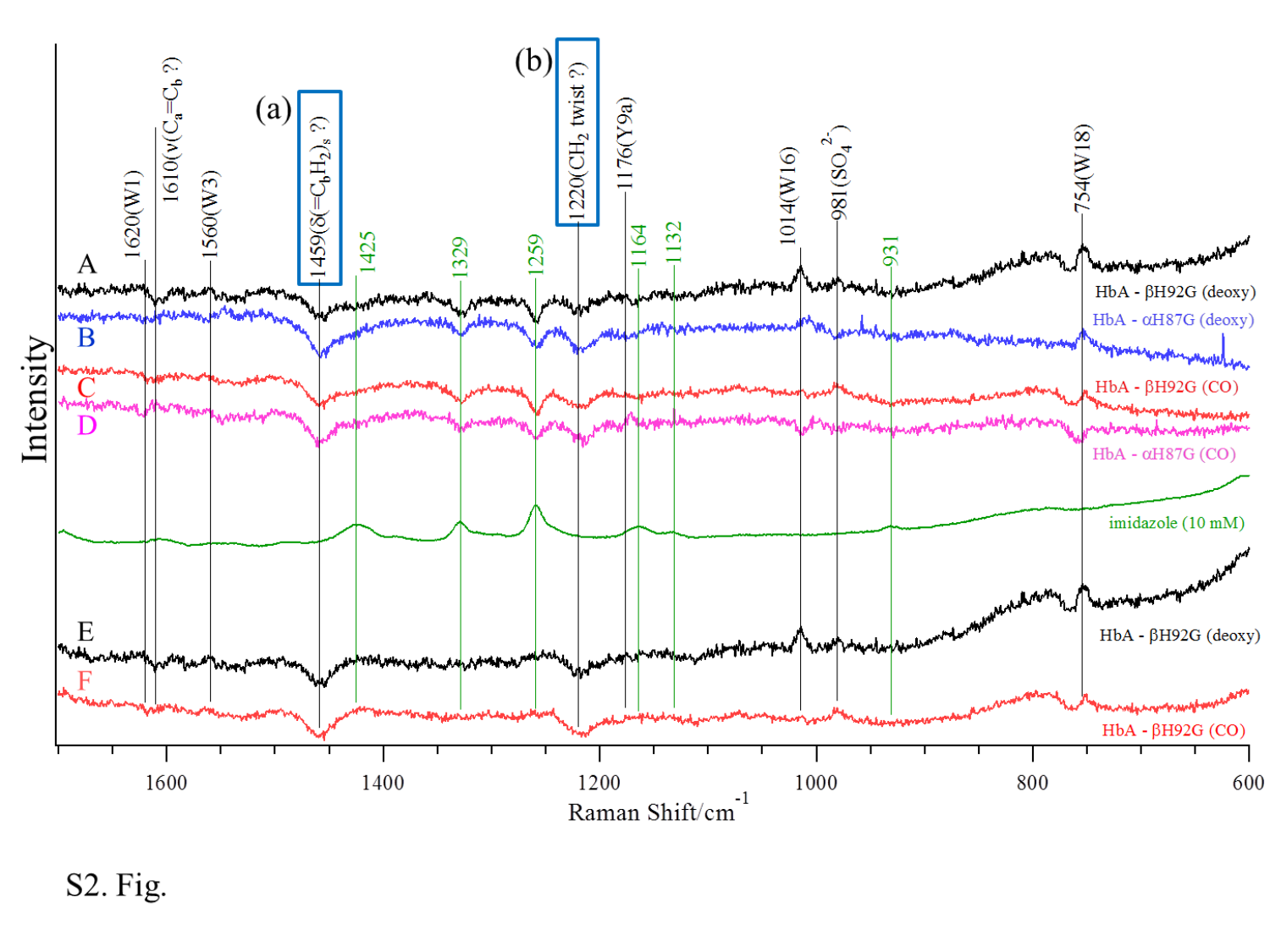

## Slide 4
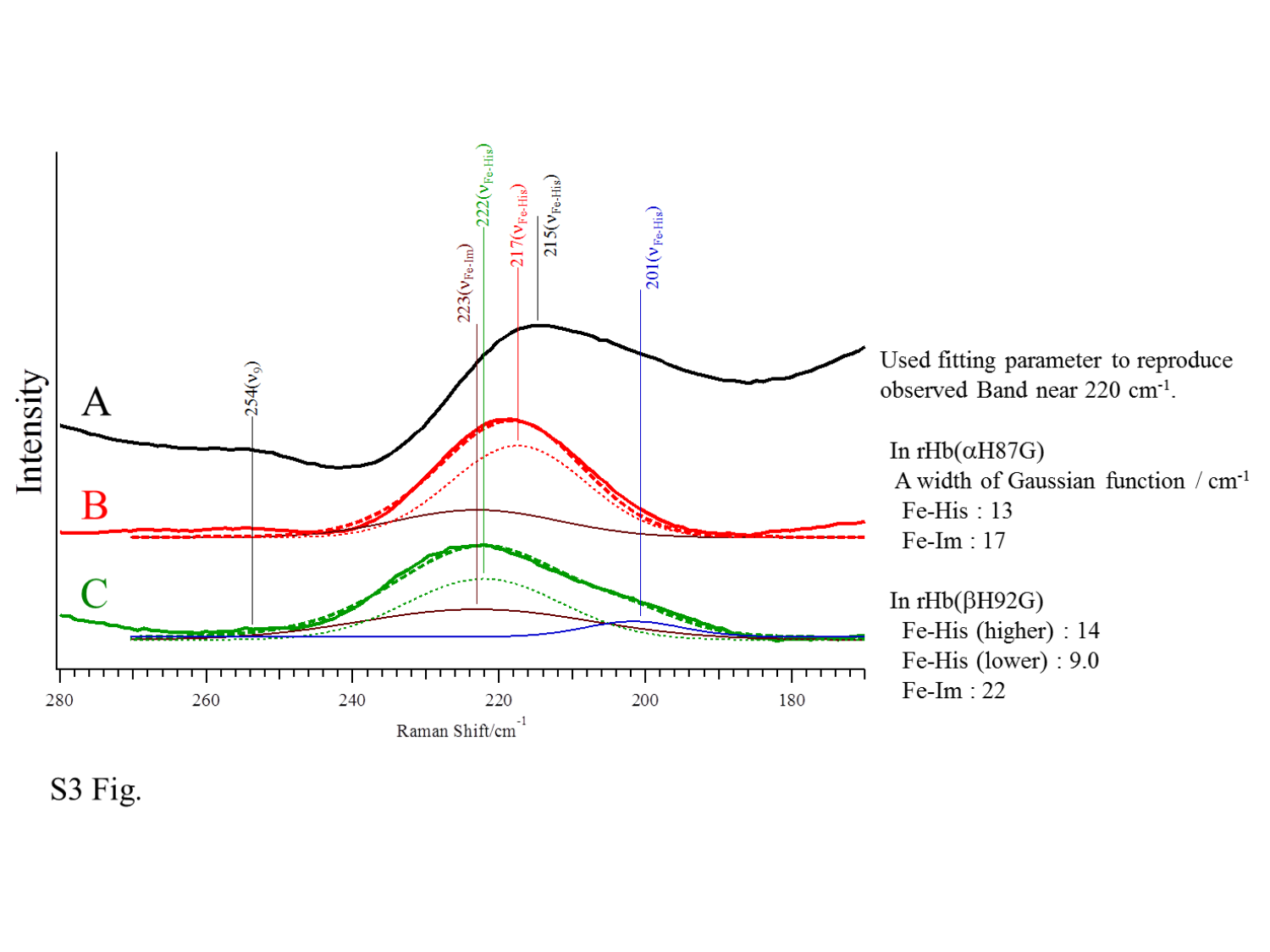

## Slide 5
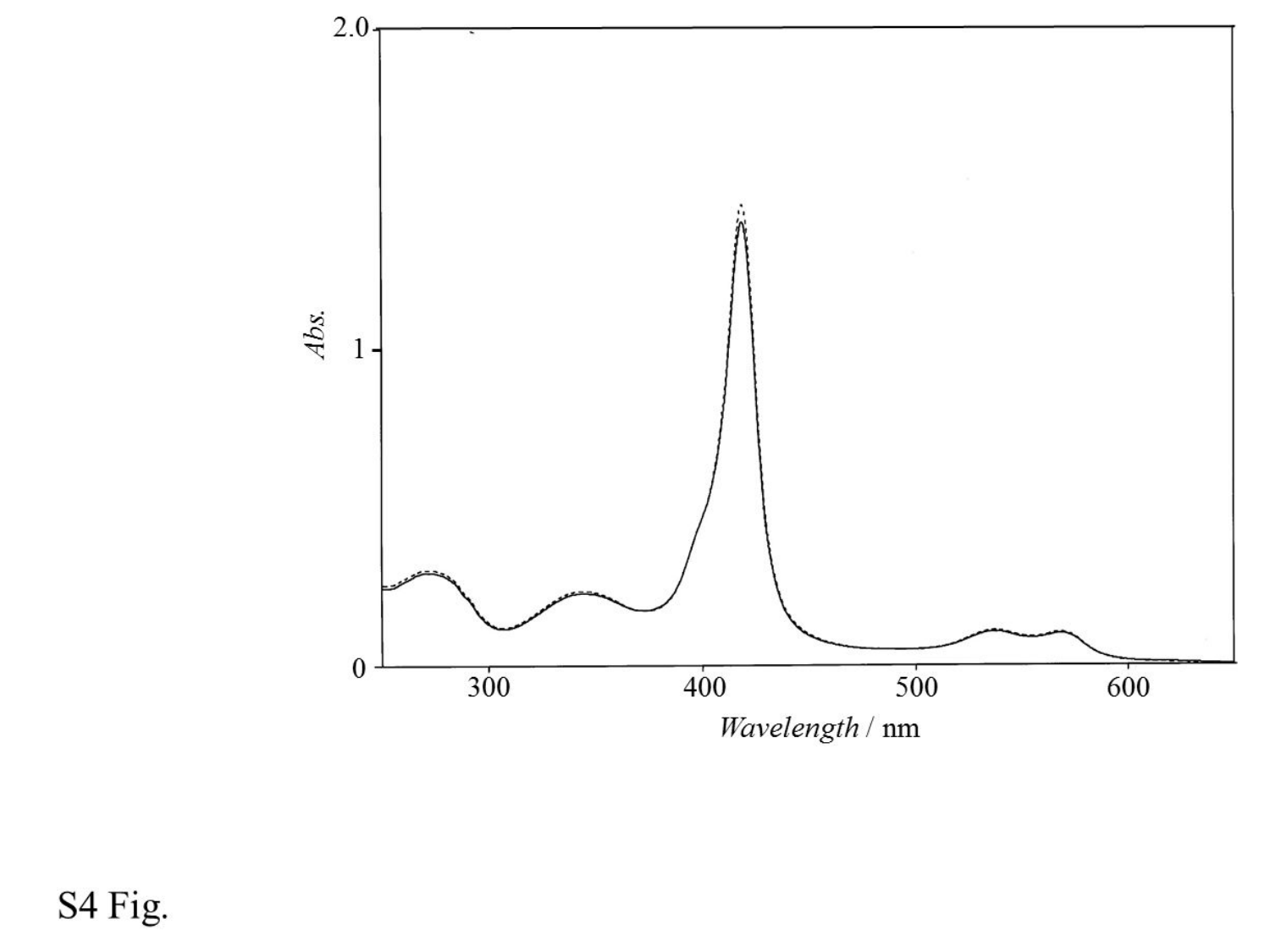

## Slide 6
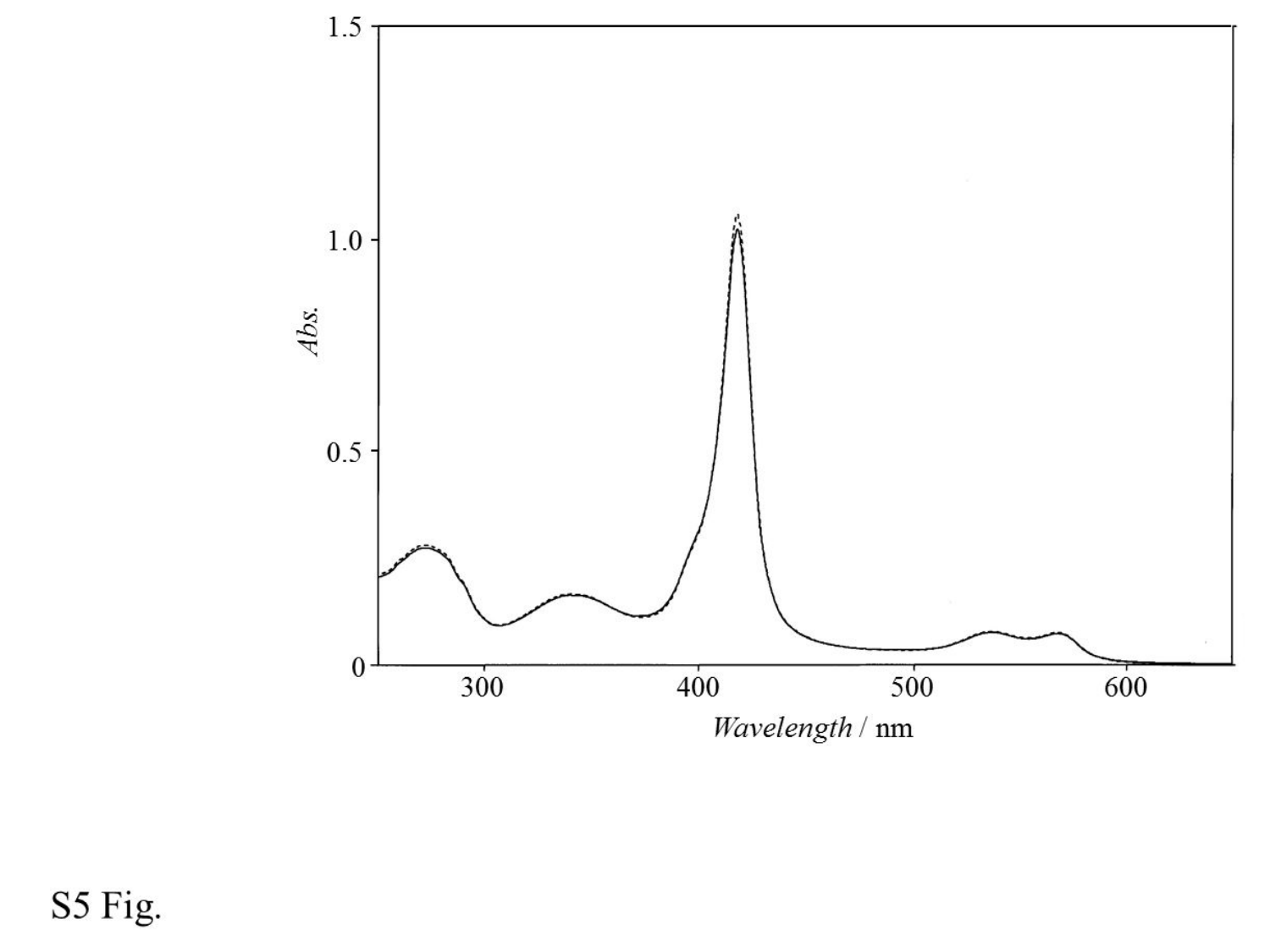

## Slide 7
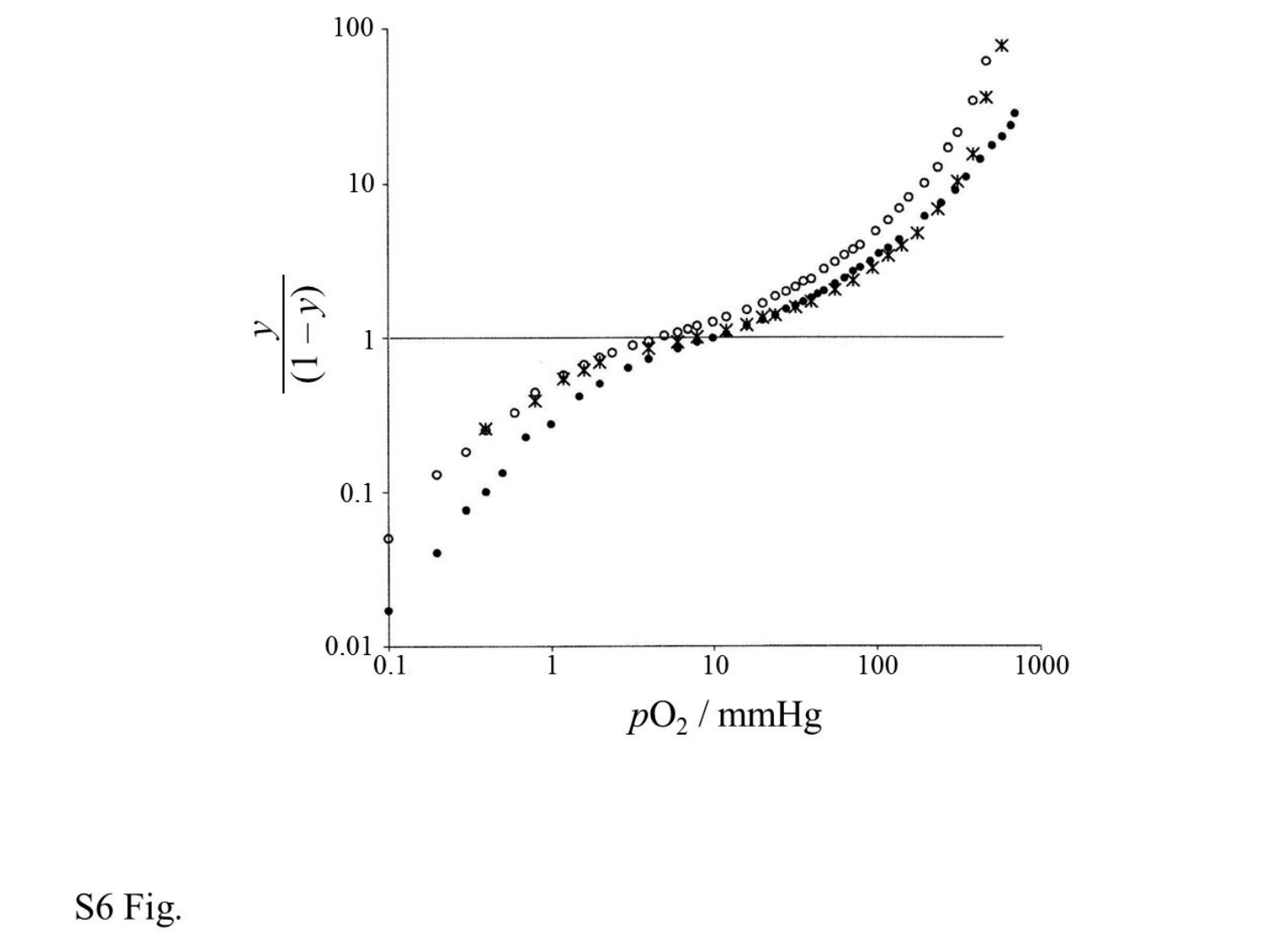

## Slide 8
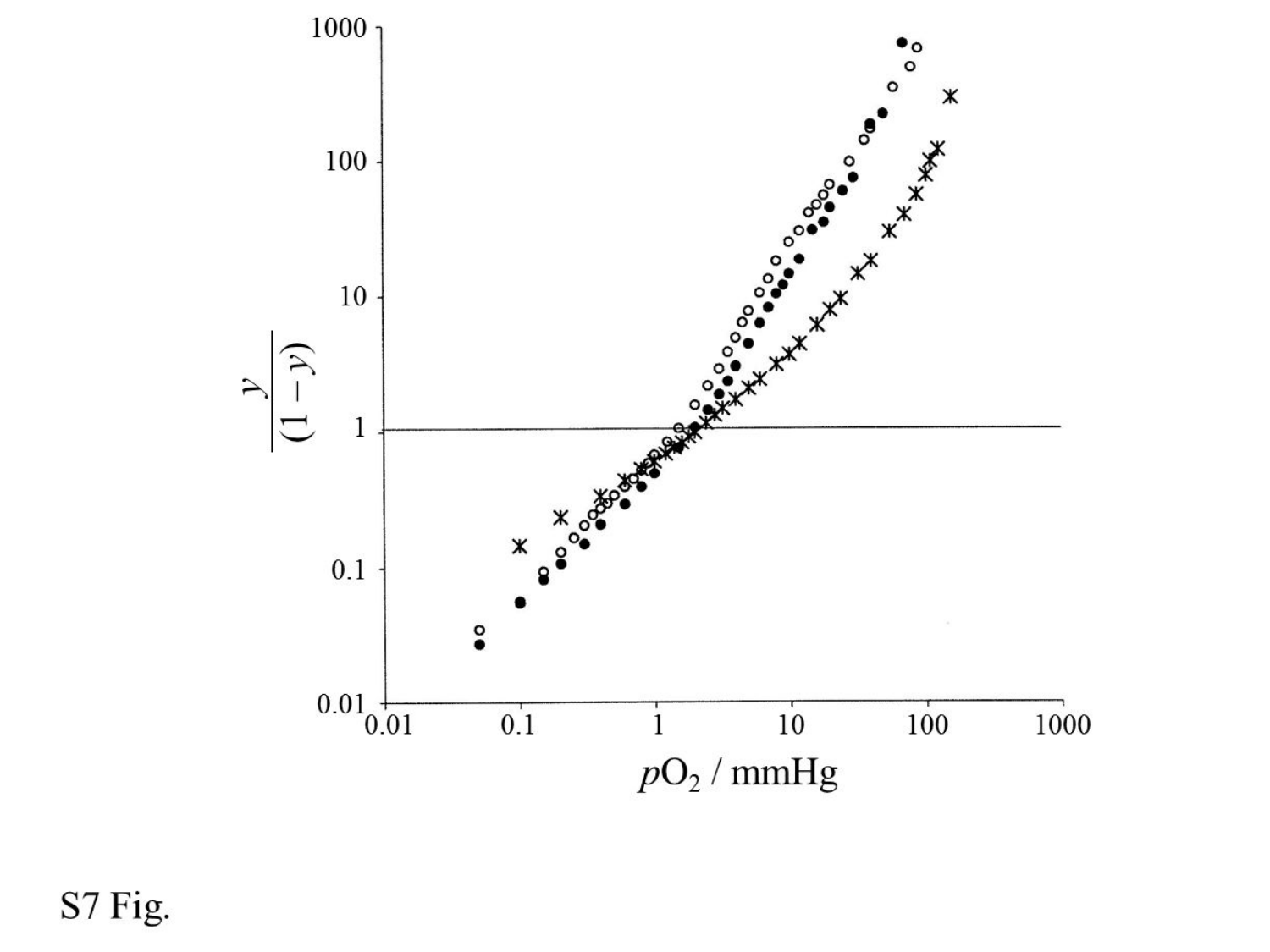

Supplement: S1 File — (PPT) [file pone.0135080.s008.ppt]
